# Effectiveness and safety of combining SGLT2 inhibitors and GLP-1 receptor agonists in individuals with type 2 diabetes: a systematic review and meta-analysis of cohort studies

**DOI:** 10.1007/s00125-025-06565-6

**Published:** 2025-10-21

**Authors:** Julia M. T. Colombijn, Jan F. de Leijer, Frank L. J. Visseren, Marianne C. Verhaar, Daniël H. van Raalte, Naveed Sattar, Robin W. M. Vernooij, Thomas T. van Sloten

**Affiliations:** 1 Department of Nephrology and Hypertension, University Medical Center Utrecht, Utrecht, the Netherlands; 2 Julius Center for Health Sciences and Primary Care, University Medical Center Utrecht, Utrecht University, Utrecht, the Netherlands; 3 Department of Vascular Medicine and Endocrinology, University Medical Center Utrecht, Utrecht, the Netherlands; 4 Diabetes Center, Department of Internal Medicine, Amsterdam University Medical Center, Amsterdam, Netherlands; 5 School of Cardiovascular and Metabolic Health, University of Glasgow, Glasgow, UK

**Keywords:** Cardiovascular disease, Combination therapy, GLP-1 RA, Heart failure, Kidney failure, Meta-analysis, Mortality, SGLT2 inhibitor, Systematic review, Type 2 diabetes

## Abstract

**Aims/hypothesis:**

Sodium–glucose cotransporter 2 (SGLT2) inhibitors and glucagon-like peptide-1 receptor agonists (GLP-1 RAs) reduce cardiorenal risk in type 2 diabetes. However, the effect of combining these drugs remains uncertain. This systematic review aimed to evaluate the potential effectiveness and safety of combination therapy compared with monotherapy in individuals with type 2 diabetes.

**Method:**

We systematically searched PubMed and Embase from inception to 1 May 2025 for cohort studies comparing the effect of combination therapy with SGLT2 inhibitor or GLP-1 RA monotherapy on (cardiovascular) mortality and cardiovascular or kidney endpoints in individuals with type 2 diabetes. Studies enrolling individuals with type 1 diabetes or a maximum follow-up of less than 1 year were excluded. The primary outcome was a composite of major adverse cardiovascular events (MACE). Secondary outcomes included all-cause mortality, cardiovascular mortality, hospitalisation for heart failure, a kidney composite endpoint and serious adverse events. Risk of bias was assessed with ROBINS-I. Risk ratios (RRs) and 95% CIs were pooled in random effects meta-analyses. Certainty of evidence was assessed using Grading of Recommendations Assessment, Development and Evaluation (GRADE).

**Results:**

We included 18 cohort studies (1,164,774 participants). In cohort studies, combination therapy was associated with a lower risk of MACE (RR 0.56 [95% CI 0.43, 0.71]; low certainty of evidence) and the kidney composite endpoint (RR 0.48 [95% CI 0.32, 0.73]; very low certainty of evidence) relative to SGLT2 inhibitor or GLP-1 RA monotherapy. Combination therapy was also associated with a lower risk of all-cause mortality (RR 0.50 [95% CI 0.40, 0.63]; low certainty of evidence), cardiovascular mortality (RR 0.26 [95% CI 0.16, 0.43]; low certainty of evidence) and hospitalisation for heart failure (RR 0.67 [95% CI 0.64, 0.71]; moderate certainty of evidence). Although safety data could not be pooled due to lack of events, no differences were observed in the risk of severe hypoglycaemia, diabetic ketoacidosis, genitourinary infections and gastrointestinal side effects. No data were reported on the risk of serious adverse events or major adverse limb events.

**Conclusions/interpretation:**

Observational studies suggest that combining an SGLT2 inhibitor and a GLP-1 RA in type 2 diabetes may lower the risk of MACE, all-cause and cardiovascular mortality, hospitalisation for heart failure and kidney composite endpoints compared with monotherapy with either drug. Of course, residual confounding cannot be overcome but results support the need for future randomised trials of combined vs monotherapy.

**Registration:**

PROSPERO registration no. CRD42024532383

**Graphical Abstract:**

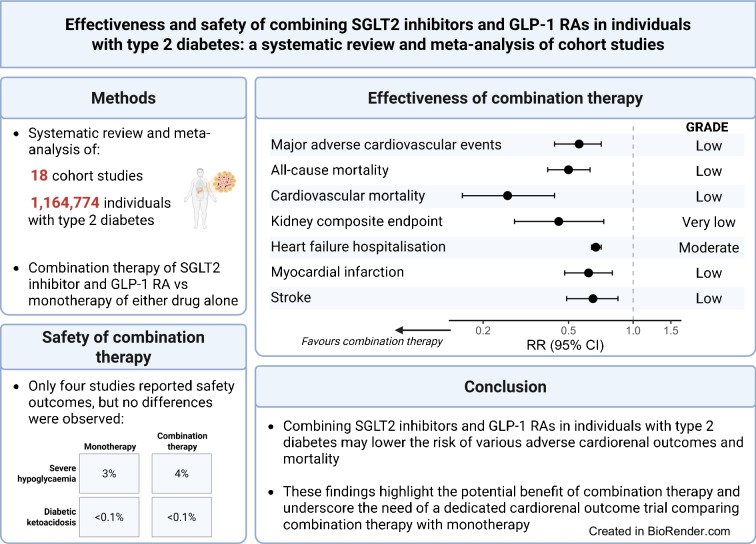

**Supplementary Information:**

The online version contains peer-reviewed but unedited supplementary material available at 10.1007/s00125-025-06565-6.


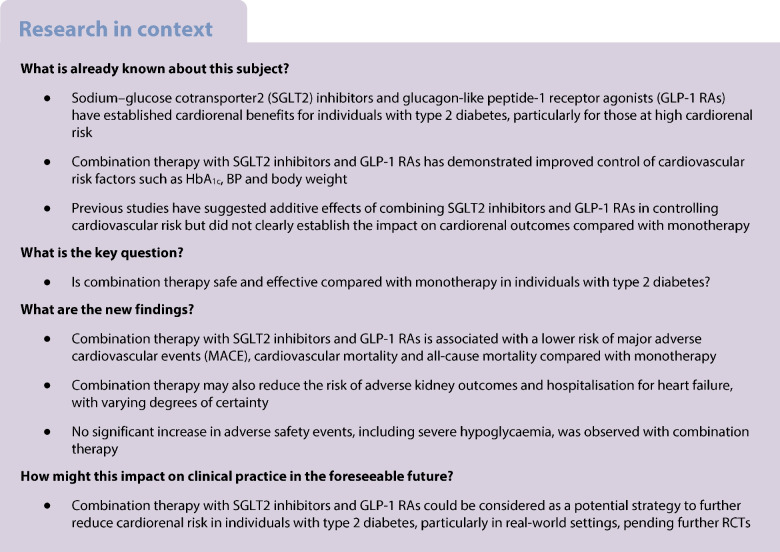


## Introduction

Type 2 diabetes affects over 800 million individuals globally. This number is projected to rise to 1.3 billion by 2050 due to an ageing population and rising prevalence of obesity [1, 2]. Individuals with type 2 diabetes have a two- to fourfold higher risk of CVD, heart failure, all-cause mortality and cardiovascular death [3, 4] and a six- to-12-fold higher risk of adverse kidney outcomes, including kidney failure [5].

Both sodium–glucose cotransporter 2 (SGLT2) inhibitors and glucagon-like peptide-1 receptor agonists (GLP-1 RAs) reduce the risk of cardiovascular events, mortality, hospitalisation for heart failure and adverse kidney outcomes in individuals with type 2 diabetes at very high cardiorenal risk (i.e. with a history of CVD, heart failure or kidney disease), independent of their glucose-lowering effects [6, 7]. Due to their cardiorenal benefits, most clinical guidelines recommend SGLT2 inhibitors and GLP-1 RAs as first-line treatment for individuals with type 2 diabetes at very high cardiorenal risk [8–12].

Recent interest in combining SGLT2 inhibitors and GLP-1 RAs has emerged due to their distinct and potentially synergistic mechanisms of action and potentially complementary effects on cardiorenal outcomes [12, 13]. While both types of drug decrease cardiorenal risk, SGLT2 inhibitors primarily reduce the risk of heart failure hospitalisation, whereas GLP-1 RAs mainly reduce the risk of stroke and myocardial infarction [14, 15]. Earlier studies have found that combination therapy with an SGLT2 inhibitor and a GLP-1 RA, compared with monotherapy with either drug alone, is associated with improved control of cardiovascular risk factors, including a greater reduction of HbA_1c_, BP and body weight, demonstrating additive effects [16–18]. However, the effect of combining these types of drugs on cardiorenal outcomes remains uncertain.

Two recent individual participant data meta-analyses of RCTs [19, 20] found that the effects of SGLT2 inhibitors and GLP-1 RAs on cardiovascular and kidney outcomes are consistent regardless of the background use of a GLP-1 RA or an SGLT2 inhibitor, respectively [19, 20]. However, neither study evaluated whether the combination therapy was more effective than monotherapy with either an SGLT2 inhibitor or a GLP-1 RA. Furthermore, the number of individuals (<10% of the total study population) and events in the combination therapy group in these meta-analyses was relatively low (e.g. <70 kidney events in the combination therapy group), resulting in a relatively large uncertainty of the observed treatment effects. Additionally, a recent narrative review of observational studies suggested that combination therapy was associated with a lower risk of major adverse cardiovascular events (MACE), cardiovascular mortality, all-cause mortality, hospitalisation for heart failure and kidney outcomes. However, this review was not performed systematically and no meta-analysis was performed [21].

To address this gap, we performed a systematic review and meta-analysis of observational studies to evaluate the potential effectiveness on cardiorenal endpoints and the safety of combining an SGLT2 inhibitor with a GLP-1 RA compared with SGLT2 inhibitor or GLP-1 RA monotherapy in individuals with type 2 diabetes. We did not include RCTs in this meta-analysis because to date only one RCT [22] has compared combination therapy with an SGLT2 inhibitor and a GLP-1 RA vs SGLT2 inhibitor or GLP-1 RA monotherapy. Other RCTs have conducted subgroup analyses on baseline SGLT2 inhibitor or GLP-1 RA use but this potentially introduced confounding due to unadjusted differences between subgroups [23].

## Methods

This meta-analysis was reported in accordance with the Preferred Reporting Items for Systematic Reviews and Meta-Analyses (PRISMA) [24]. The protocol was registered a priori in PROSPERO (registration no. CRD42024532383).

### Search strategy and selection criteria

We systematically searched MEDLINE and Embase from inception to 1 May 2025 using a combination of search terms related to diabetes, SGLT2 inhibitors and GLP-1 RAs. The complete search strategy is detailed in electronic supplementary material (ESM) Tables 1 and 2. Additional studies were identified by screening the reference lists of included studies. Two reviewers (JC and JdL) independently screened records at the title, abstract and full text level. Discrepancies were resolved by a third reviewer (TvS).

Eligible studies were cohort studies in individuals with type 2 diabetes that evaluated the effects of a combination of an SGLT2 inhibitor and a GLP-1 RA in comparison with monotherapy with either an SGLT2 inhibitor or a GLP-1 RA on (cardiovascular) mortality and cardiovascular or kidney endpoints. We excluded studies enrolling individuals with type 1 diabetes and studies with a maximum follow-up period of less than 1 year. No restrictions on language or publication status were applied.

### Data collection and items

We extracted study characteristics, participant characteristics and relevant outcomes of included studies using a standardised extraction form. One reviewer performed the data extraction, which was independently verified by a second reviewer (JC or JdL). The authors of two included studies were contacted for additional information but none responded to our enquiries [25, 26].

### Outcomes

The primary outcome was a composite of MACE as defined by the study investigators. Secondary outcomes included all-cause mortality, cardiovascular mortality, non-fatal myocardial infarction, non-fatal stroke, hospitalisation for heart failure, and adverse kidney outcomes (i.e. dialysis, kidney transplantation, a sustained eGFR <15 ml/min per 1.73 m^2^, doubling of serum creatinine, kidney death or adverse kidney outcomes as defined by the study investigators). Safety outcomes included any serious adverse events, severe hypoglycaemia, diabetic ketoacidosis, genitourinary infections, major adverse limb events and gastrointestinal side effects.

### Risk of bias assessment

The risk of bias assessment was performed with ROBINS-I for cohort studies [27]. Two reviewers independently evaluated risk of bias of included studies (JC and JdL), with any disagreements resolved by a third reviewer (TvS).

### Data synthesis

Study and participant characteristics were summarised using descriptive statistics. Outcome measures were pooled in a random effects meta-analysis. Effect estimates were expressed as risk ratios (RRs) and corresponding 95% CIs. In the main analysis, the monotherapy arms of SGLT2 inhibitor or GLP-1 RA use were pooled as a single control group.

Heterogeneity was analysed using the Cochran *Q* test and the *I*^2^ statistic. Subgroup analyses on MACE were performed according to the type of the control intervention (SGLT2 inhibitor or GLP-1 RA monotherapy), mean baseline HbA_1c_ (>64 mmol/mol [>8%] vs ≤64 mmol/mol [≤8%]), mean baseline diabetes duration (>10 years vs ≤10 years) and the presence vs absence of prior CVD, chronic kidney disease (CKD) or heart failure. Sensitivity analyses were performed to assess the impact of the definition of MACE (three-point MACE [non-fatal myocardial infarction, non-fatal stroke and cardiovascular death] vs other MACE definitions), risk of bias, data source (electronic healthcare records vs other data sources) and method of adjusting for confounding (propensity scores or inverse probability of treatment weighting vs multivariable regression, restriction or matching). Heterogeneity between subgroups was formally tested using interaction terms. Publication bias was evaluated with funnel plots. A *p* value and *p* value for interaction (*p*_interaction_) <0.05 were considered statistically significant. All analyses were performed in R version 4.4.2 (R Development Core Team, Vienna, Austria). The dataset and syntax are available as separate ESMs (ESM Dataset and ESM Syntax).

### Certainty of evidence

The certainty of evidence was assessed with the Grading of Recommendations Assessment, Development and Evaluation (GRADE) approach [28]. Two authors independently applied the GRADE criteria for each outcome (JC and JdL), with any disagreements resolved by a third reviewer (TvS).

## Results

After removing duplicates, 4530 study reports were screened for eligibility. Eighteen studies comprising 1,164,774 participants were included [25, 26, 29–44]. Of these, 15 studies (991,731 participants) were eligible for the meta-analyses [29–43]. The three other studies were ineligible because two did not report the number of events and the authors did not respond to our request to provide the data [25, 26] and one was a nested case–control study [44]. The study selection process and a list of excluded studies are provided in ESM Fig. 1 and ESM Table 3. A detailed overview of the study and participant characteristics, baseline medication use, key methodology, eligibility criteria and outcomes and risk of bias assessment for the individual studies is presented in Table 1, ESM Tables 4–7 and ESM Fig. 2.

**Table 1:** Study and baseline characteristics of included cohort studies

Study (publication year)	Comparison (*n*)	Location	Follow-up (months)	Funding	Total sample size	Age (years)	Sex female (*N*, %)	HbA_1c_ (mmol/mol, %)	Diabetes duration (years)	CVD (%)	Heart failure (*n*, %)	CKD (*n*, %)	eGFR (ml/min per 1.73 m^2^)	UACR (mg/mmol)	BP or SBP/ DBP (mmHg)	BMI (kg/m^2^)
Chaiyakunapruk et al (2025) [29]	GLP-1 RA + SGLT2-i (34,690) vs SGLT2-i (130,220)	USA	>6	Novo Nordisk	164,910	64 ± 11	60,370 (37)	64 ± 14 (8.0 ± 1.7)	-	164,910 (100)	35,134 (21)	2586 (2)	-	-	-	32 ± 6
Dave et al (2021) [30]	SGLT2-i + GLP-1-RA (12584) vs sulfonylurea + GLP1-RA (12584)	USA	10	Division of Pharmacoepidemiology and Pharmacoeconomics, Brigham and Women’s Hospital	25,168	58 ± 11	13,050 (52)	68 ± 15 (8.4 ± 1.8)	-	5156 (20)	836 (3)	1864 (7)	-	-	-	-
García-Vega et al (2024) [25]	GLP-1 RA + SGLT2-i (2449) vs SGLT2-i (12,029)	Spain	18	No funding reported	15,549	69 ± 11	6433 (41)	-	-	CAD 2353 (15) Stroke 913 (6)	1470 (9)	-	-	-	-	-
Gorgojo-Martínez et al (2017) [31]	Dapaglifozin + GLP-1 RA (109) vs dapagliflozin (104)	Spain	12	No funding reported	213	59 ± 11	99 (47)	57 ± 10 (7.4 ± 1.3)	11 (6–17)	CAD 31 (15) Stroke 14 (7) PAD 18 (9)	-	45 (21)	93 ± 25	-	140 ± 14 / 76 ± 9	35 ± 6
Horiuchi et al 2025 [26]	GLP-1 RA + SGLT2-i (3518) vs SGLT2-i (3518)	Japan	36	No funding reported	131,196	-	-	-	-	-	-	-	-	-	-	-
Jensen et al (2020) [32]	GLP-1 RA + SGLT2-i (1823) vs GLP-1 RA (6515) vs SGLT2-i (8326)	Denmark	Up to 240	No funding reported	16,664	59 ± 11	6951 (42)	-	7 ± 5	1305 (8)	-	181 (1)	-	-	-	-
Jhu et al (2025) [33]	GLP-1 RA + SGLT2-i (71,186) vs SGLT2-i (71,186)^a^	Multinational	Up to 60	No funding reported	142,372	57 ± 11	64,470 (45)	66 ± 15 (8.2 ± 1.9)	-	CAD 28,998 (20) Stroke 12,762 (9) PAD 6884 (5)	14,887 (10)	-	88 ± 17	-	-	34 ± 20
Kobayashi et al (2023) [34]	SGLT2-i + GLP-1 RA (186) vs GLP-1 RA (186)	Japan	47 [26–68]	No funding reported	372	63 ± 11	172 (46)	68 ± 13 (8.4 ± 1.6)	-	-	-	-	74 ± 26	3.2 [1.2–12.4]	132 ± 17	-
Lau et al (2022) [35]	Add on of GLP-1 RA or DPP-4i to SGLT2-i (1461) vs switch from SGLT2-i to GLP-1 RA or DPP-4i (1427)	UK	18 [9–32]	No funding reported	2888	58 ± 11	1337 (46)	75 ± 12 (9.0 ± 1.5)	9 ± 6	549 (19)	72 (3)	566 (20)	114 ± 30	6.6 ± 29	132 ± 14 / 78 ± 9	35 ± 7
Liu et al (2025) [36]	GLP-1 RA + SGLT2-i (208) vs SGLT2-i (208)	China	12	Civil Aviation General Hospital	416	63 ± 10	134 (32)	57 ± 24 (7.4 ± 3.1)	8 ± 3	416 (100)	67 (16)	-	-	14.5 ± 4.2	-	25 ± 8
Lopez et al (2022) [37]	GLP-1 RA + SGLT2-i (343) vs SGLT2-I (343)	USA	36	No funding reported	686	68 ± 8	6 (1)	64 ± 14 (8.0 ± 1.7)	-	686 (100)	686 (100)	-	55 ± 23	-	-	33 ± 7
Luo et al (2023) [38]	GLP-1 RA + SGLT2-i (1116) vs GLP-1 RA (558)	Hong-Kong	-	No funding reported	1674	53 ± 11	696 (42)	70 ± 16 (8.6 ± 2.0)	8 ± 5	CAD 130 (8) Stroke/TIA 21 (1)	48 (3)	108 (7)	93 ± 27	-	-	-
Marfella et al (2024) [39]	Start SGLT2-i or GLP-1 RA to receive combination therapy (214) vs SGLT-2i (99) vs GLP-1 RA (130)	Italy	9 ± 3	Programmi di Ricerca Scientifica di Rilevante Interesse Nazionale (Scientific research programs of high national interest)	443	69 (63–73)	180 (41)	60 [54–66] (7.6 [7.1–8.2])	15 (13–17)	443 (100)	-	-	-	-	-	28 (27–29)
Patel et al (2024) [40]	GLP-1 RA + SGLT2-i (7044) vs SGLT2-i (7044)	USA	12	Individual authors have disclosed several funding sources from, among others, various pharmaceutical companies	14,088	63 ± 11	6175 (44)	72% withHbA_1c_ >53 mmol/mol (>7%)	-	CAD 7734 (55) Stroke 1213 (9) PAD 3811 (27)	14088 (100)	4124 (29)	-	-	-	36 ± 6
Riley et al (2023) [41]	GLP-1 RA + SGLT2-i (107,643) vs GLP-1 RA (107,643) vs SGLT2-i (96,291)^a^	Multinational	60	European Union’s Horizon 2020 research and innovation programme, honoraria from Procter and Gamble, Viatris and Sanofi	311,577	60	149,274 (48)	59% with HbA_1c_ >53 mmol/mol (>7%)	-	-	49,363 (16)	63,112 (20)	-	-	-	-
Schechter et al (2023) [42]	GLP-1 RA + SGLT2-i (475) vs GLP-1 RA (2949) vs SGLT2-i + insulin (475)	Israel	81 [51–110]	Novo Nordisk	6848	59 ± 10	3067 (45)	75 ± 12 (9.0 ± 1.5)	10 ± 5	1438 (21)	-	-	91 ± 19	1.7 [0–6.2]	-	33 ± 5
Simms-Williams et al (2024a) [43]	GLP-1 RA + SGLT2-i (6696) vs GLP-1 RA (6696)	UK	9	Foundation Scheme grant from the Canadian Institutes of Health Research	13,392	57 ± 10	6097 (46)	80% with HbA_1c_ >64 mmol/mol (>8%)	11 ± 6	3686 (28)	373 (3)	1414 (11)	-	-	-	86% with BMI >30
Simms-Williams et al (2024b) [43]	GLP-1 RA + SGLT2-i (8942) vs SGLT2-i (8942)	UK	9	Foundation Scheme grant from the Canadian Institutes of Health Research	17,884	58 ± 10	8456 (47)	78% with HbA_1c_ >64 mmol/mol (>8%	10 ± 5	4826 (27)	802 (4)	1637 (9)	-	-	-	81% with BMI >30
Wright et al (2022a) [44]	GLP-1 RA + SGLT2-i (49) vs GLP-1 RA (372) vs SGLT2-i (643)	UK	40	Diabetes UK, Medical Research Council Health eResearch Centre grant and methodology award	1,064	60 ± 9	728 (68)	77 ± 14 (9.0 ± 1.5)	5 ± 5	0	-	9 (1)	-	-	-	35 ± 19
Wright et al (2022b) [44]	GLP-1 RA + SGLT2-i (793) vs GLP-1 RA (5851) vs SGLT2-i (8779)	UK	48	Diabetes UK, Medical Research Council Health eResearch Centre grant and methodology award	15,423	61 ± 9	9841 (64)	75 ± 14 (9 ± 1.7)	6 ± 5	0	-	1342 (9)	-	-	-	35 ± 6
Wright et al (2022c) [44]	GLP-1 RA + SGLT2-i (433) vs GLP-1 RA (2748) vs SGLT2-i (3678)	UK	48	Diabetes UK, Medical Research Council Health eResearch Centre grant and methodology award	6859	60 ± 9	4369 (64)	79 ± 14 (9.4 ± 1.7)	9 ± 4	0	-	5816 (85)	-	-	-	35 ± 6

Continuous variables are presented as mean ± SD or median (IQR) and categorical variables as *n* (%)

^a^Sum may not equal total number of participants included in the meta-analysis since the number of participants at risk for each outcome varied (Jhu) or because they were excluded if they had experienced the outcome prior to the index date (Riley)

CAD, coronary artery disease; DBP, diastolic BP; PAD, peripheral arterial disease; SBP, systolic BP; SGLT2-i, SGLT2 inhibitor; TIA, transient ischaemic attack; UACR, urinary albumin creatinine ratio

Follow-up ranged from 8 months to 240 months. Fifteen studies [25, 26, 29, 31–34, 36–38, 40–44] evaluated the addition of an SGLT2 inhibitor or GLP-1 RA to background GLP-1 RA or SGLT2 inhibitor therapy. One study [30] compared adding an SGLT2 inhibitor or sulfonylurea to GLP-1 RA therapy; another [35] compared adding a GLP-1 RA or a dipeptidyl peptidase-4 (DPP-4) inhibitor to an SGLT2 inhibitor with switching from a SGLT2 inhibitor to a GLP-1 RA or DPP-4 inhibitor; and one study [39] compared adding an SGLT2 inhibitor or GLP-1 RA (if HbA_1c_ >53 mmol/mol) to continuing monotherapy (if HbA_1c_ ≤53 mmol/mol). Participants’ mean age was 61 ± SD 11 years, 33% were female, mean baseline HbA_1c_ was 65 ± SD 14 mmol/mol (8.1 ± 1.8%) and mean diabetes duration was 9 ± SD 5 years (Table 1 provides data for the individual studies). Sixteen of the 18 included cohort studies had severe or critical methodological limitations, primarily due to suboptimal (reporting of) adjustment for potential confounders and poor handling of missing data (ESM Tables 6, 7, ESM Fig. 2).

### Association between SGLT2 inhibitor and GLP-1 RA combination therapy and cardiorenal endpoints

**Fig. 1:**
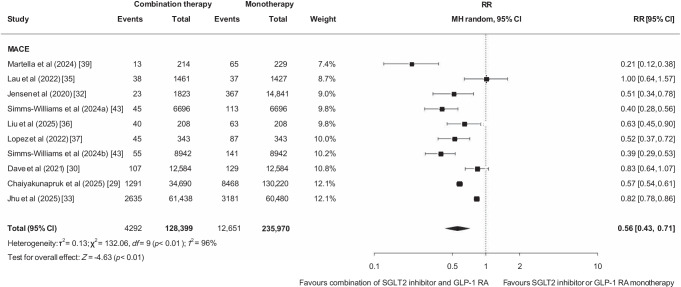
Effect of combining an SGLT2 inhibitor and a GLP-1 RA compared with SGLT2 inhibitor or GLP-1 RA monotherapy on MACE in cohort studies. MH, Mantel–Haenszel

Ten studies (*n*=387,715) [29, 30, 32, 33, 35–37, 39, 43, 44] reported on MACE. Among the nine studies included in the pooled analysis (*n*=364,369) [29, 30, 32, 33, 35–37, 39, 43], 16,943 events were observed (5%). Compared with monotherapy, combining SGLT2 inhibitors and GLP-1 RAs was associated with a lower risk of MACE (RR 0.56 [95% CI 0.43, 0.71]; low certainty of evidence due to risk of bias and inconsistency; Fig. 1, Table 2). The nested case–control study not included in the pooled analysis also reported a lower risk of MACE for combination therapy (adjusted OR [aOR] 0.70 [95% CI 0.50, 0.98]) [44].

**Table 2:** Summary of findings: Combining a SGLT2 inhibitor and GLP-1 receptor agonist in individuals with type 2 diabetes

Outcome	No. of participants (studies)	Relative effect (95% CI)	Anticipated absolute effects (95% CI)		Certainty (GRADE)
			Monotherapy	Combination therapy	
MACE	364,369 (9)	0.56 (0.43, 0.71)	54 / 1000	30 / 1000 (23, 38)	⊕⊕⊝⊝^a,b^
All-cause mortality	542,989 (10)	0.50 (0.40, 0.63)	38 / 1000	19 / 1000 (1, 24)	⊕⊕⊝⊝^a,b^
Cardiovascular mortality	31,692 (2)	0.26 (0.16, 0.43)	5 / 1000	1 / 1000 (1, 2)	⊕⊕⊝⊝^a,c^
Kidney composite endpoint	299,583 (6)	0.48 (0.32, 0.73)	77 / 1000	35 / 1000 (22, 56)	⊕⊝⊝⊝^a,b,d^
Hospitalisation for heart failure	43,246 (5)	0.67 (0.64, 0.71)	101 / 1000	68 / 1000 (65, 72)	⊕⊕⊕⊝^a^
Myocardial infarction	508,018 (6)	0.62 (0.48, 0.80)	22 / 1000	14 / 1000 (11, 18)	⊕⊕⊝⊝^a,b^
Stroke	509,116 (6)	0.65 (0.49, 0.86)	23 / 1000	15 / 1000 (11, 20)	⊕⊕⊝⊝^a,b^

^a^Evidence certainty was downgraded one level due to risk of bias

^b^Evidence certainty was downgraded one level due to inconsistency (heterogeneity in point estimates with *I*^2^ up to 96%)

^c^Evidence certainty was downgraded one level due to imprecision (two included studies with only 101 events in total)

^d^Evidence certainty was downgraded one level due to indirectness (all studies used surrogate endpoints for kidney failure)

**Fig. 2:**
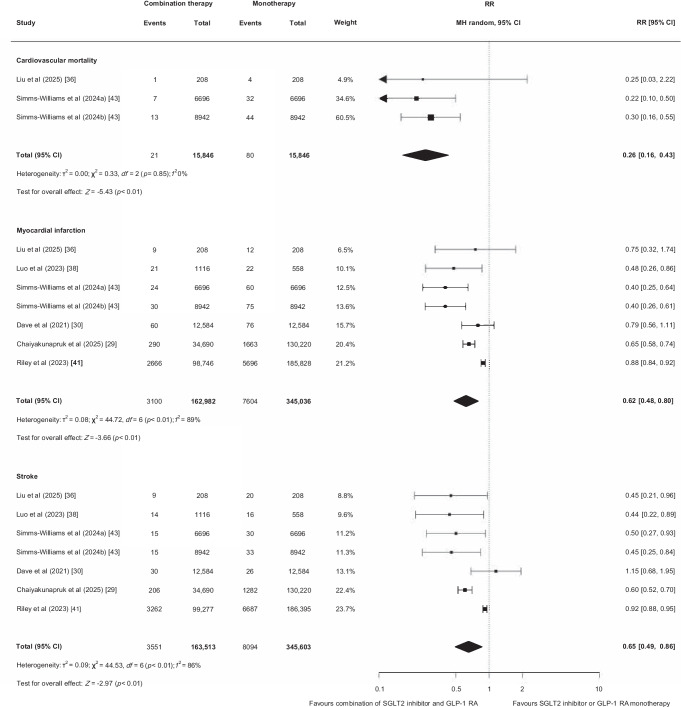
Effect of combining an SGLT2 inhibitor and a GLP-1 RA compared with SGLT2 inhibitor or GLP-1 RA monotherapy on cardiovascular mortality, myocardial infarction and stroke in cohort studies. MH, Mantel–Haenszel

Three studies (*n*=57,738) reported cardiovascular mortality [36, 43, 44]. Among the two included in the pooled analysis (*n*=31,692), 101 events were recorded (0.3%) [36, 43]. Compared with SGLT2 inhibitor or GLP-1 RA monotherapy, combination therapy was associated with a lower risk of cardiovascular mortality (RR 0.26 [95% CI 0.16, 0.43]; low certainty of evidence due to risk of bias and imprecision; Fig. 2, Table 2). The nested case–control study not included in the pooled analysis did not observe a lower risk of cardiovascular mortality (aOR 0.35 [95% CI 0.08, 1.53]) [44].

Nine studies reported myocardial infarction (*n*=547,499) and stroke (*n*=548,597) [25, 29, 30, 36–38, 41, 43, 44]. Among the six studies included in the pooled analysis (myocardial infarction: *n*=508,018; stroke: *n*=509,116), 10,704 myocardial infarctions (2%) and 11,645 strokes were observed (2%) [29, 30, 36, 38, 41, 43]. Compared with monotherapy, combination therapy with an SGLT2 inhibitor and a GLP-1 RA was associated with a lower risk of myocardial infarction (RR 0.62 [95% CI 0.48, 0.80], low certainty of evidence due to risk of bias and inconsistency; Fig. 2, Table 2) and stroke (RR 0.65 [95% CI 0.49, 0.86], low certainty of evidence due to risk of bias and inconsistency; Fig. 2, Table 2). Results of the studies not included in the pooled analysis were inconsistent. None of these studies reported a lower risk of myocardial infarction. Reported estimates were as follows: García-Vega et al *p*=0.15 [25]; Wright et al aOR 0.66 (95% CI 0.43, 1.04) [44]; and Lopez et al HR 0.36 (95% CI 0.11, 1.07) [37]. Only García-Vega et al [25] observed a lower risk of stroke (*p*=0.005) but Wright et al [44] (aOR 0.81 [95% CI 0.52, 1.27]) and Lopez et al [37] (HR 1.03 [95% CI 0.27, 4.36]) did not find a reduction.

**Fig. 3:**
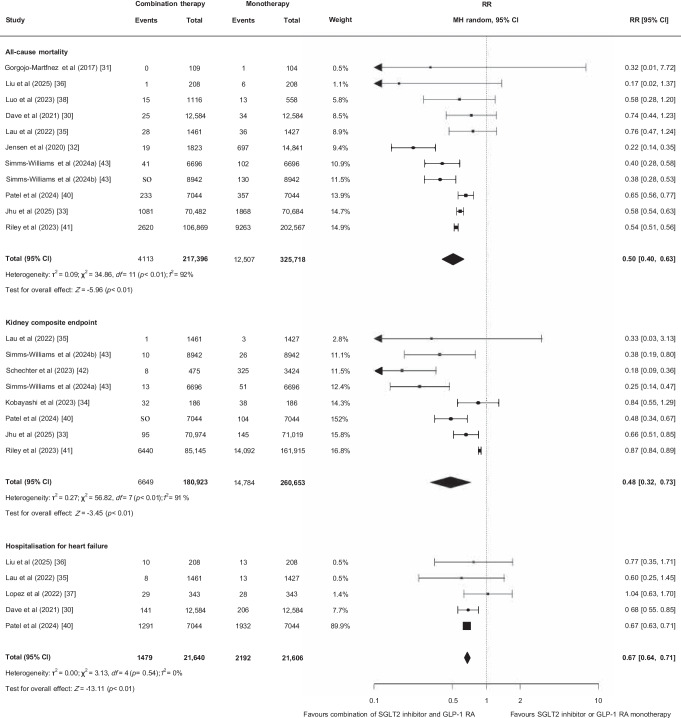
Effect of combining an SGLT2 inhibitor and a GLP-1 RA compared with SGLT2 inhibitor or GLP-1 RA monotherapy on all-cause mortality, the kidney composite endpoint and hospitalisation for heart failure. MH, Mantel–Haenszel

Twelve studies reported all-cause mortality (*n*=581,884) [25, 30–33, 35, 36, 38, 40, 41, 43, 44]. Among the ten studies included in the pooled analysis (*n*=542,989), 16,620 deaths were reported [30–33, 35, 36, 38, 40, 41, 43]. Compared with SGLT2 inhibitor or GLP-1 RA monotherapy, combination therapy was associated with a lower risk of all-cause mortality (RR 0.50 [95% CI 0.40, 0.63]; low certainty of evidence due to risk of bias and inconsistency; Fig. 3, Table 2). A lower risk of mortality was also observed in the studies not included in the pooled analysis (HR 0.32 [95% CI 0.25, 0.41]; aOR 0.43 [95% CI 0.28, 0.64]) [25, 44].

Seven studies [33–35, 40–43] reported adverse kidney outcomes (*n*=441,576), with 21,433 participants reaching the kidney composite endpoint (5%). Seven studies reported (hospitalisation for) heart failure (*n*=82,141) [25, 30, 35–37, 40, 44]. Among the five studies included in the pooled analysis (*n*=43,246), 3671 of 43,246 (8%) participants were hospitalised for heart failure [30, 35–37, 40]. Compared with monotherapy, combination therapy with an SGLT2 inhibitor and a GLP-1 RA was associated with a lower risk of the kidney composite endpoint (RR 0.48 [95% CI 0.32, 0.73]; very low certainty of evidence due to risk of bias, inconsistency and indirectness; Fig. 3, Table 2) and hospitalisation for heart failure (RR 0.67 [95% CI 0.64, 0.71]; moderate certainty of evidence due to risk of bias; Fig. 3, Table 2). The two studies not included in the pooled analysis also reported a lower risk for hospitalisation for heart failure (HR 0.77 [95% CI 0.61, 0.97]; aOR 0.43 [95 %CI 0.28, 0.64]) [25, 44]. One study reported a composite outcome of mortality and hospitalisation for heart failure (*n*=131,196) and found a reduced risk with combination therapy (HR 0.77 [95% CI 0.69, 0.86]) [26].

### Safety of combining SGLT2 inhibitors and GLP-1 RAs

Safety outcomes could not be pooled due to insufficient data. However, no increased risk of adverse events was observed. Four studies [31, 33, 35, 40] reported severe hypoglycaemia, with 3075 events observed among the 70,828 (4%) participants in the combination therapy group compared with 2284 events among the 72,657 (3%) participants in the monotherapy group (ESM Table 8). Three studies [31, 33, 35] reported diabetic ketoacidosis, with 263 events observed among the 69,786 (<1%) participants on combination therapy compared with 261 events among the 69,692 (<1%) participants on monotherapy (ESM Table 9). No differences were observed in the risk of genitourinary tract infections or gastrointestinal side effects between the treatment groups (ESM Tables 10, 11). No studies reported serious adverse events or major adverse limb events.

### Heterogeneity in treatment effects

**Fig. 4:**
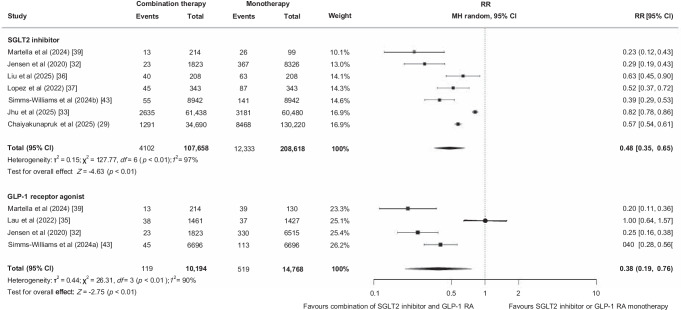
Effect of combining an SGLT2 inhibitor and a GLP-1 RA compared with SGLT2 inhibitor or GLP-1 RA monotherapy on MACE stratified by control intervention. MH, Mantel–Haenszel

The benefit of combination therapy on MACE was stronger in studies that included participants with a longer diabetes duration (>10 years) than in studies that included participants with a shorter duration (≤10 years) (*p*_interaction_ <0.01; ESM Fig. 3). No heterogeneity was observed by type of control intervention (SGLT2 inhibitor vs GLP-1 RA), baseline history of CVD, CKD or heart failure, or baseline HbA_1c_ (>64 mmol/mol vs ≤64 mmol/mol) (Fig. 4 and ESM Figs 4, 5). Additionally, results were consistent across studies with different MACE definitions (three-point vs other MACE), risk of bias level (low vs some concerns/high risk of bias), different methods to adjust for confounding, and data sources (ESM Figs 6–9).

## Discussion

This systematic review and meta-analysis of 18 cohort studies, encompassing over 1,000,000 individuals with type 2 diabetes, evaluated the potential effectiveness and safety of combining SGLT2 inhibitors and GLP-1 RAs compared with monotherapy with either an SGLT2 inhibitor or a GLP-1 RA. Our results provide some support for the combined use of SGLT2 inhibitors and GLP-1 RAs to further reduce the cardiorenal risk in individuals with type 2 diabetes, based on real-world evidence. Combination therapy was associated with a lower risk of MACE, cardiovascular mortality, the kidney composite endpoint, myocardial infarction and stroke. Combination therapy also likely lowers the risk of all-cause mortality and hospitalisation for heart failure.

Our findings confirm the results of three previous meta-analyses [19–21, 45] that evaluated the effect of combination therapy with an SGLT2 inhibitor and a GLP-1 RA on cardiovascular and kidney outcomes in individuals with type 2 diabetes. Apperloo et al [19] and Neuen et al [20] evaluated whether the benefits of SGLT2 inhibitors and GLP-1 RAs are consistent in individuals receiving or not receiving GLP-1 RAs or SGLT2 inhibitors, respectively. They found that the effects of these drugs on cardiovascular and kidney outcomes were consistent regardless of the background use of GLP-1 RAs or SGLT2 inhibitors [19, 20]. However, neither study evaluated whether the combination therapy was more effective than monotherapy with either an SGLT2 inhibitor or a GLP-1 RA. A recent narrative review of observational studies [21] also suggested that combination therapy was associated with a lower risk of MACE, all-cause mortality, hospitalisation for heart failure, and kidney failure. Another meta-analysis [45] suggested that SGLT2 inhibitor and GLP-1 RA combination therapy was associated with a lower risk of MACE, a composite outcome of hospitalisation for heart failure, cardiovascular mortality and all-cause mortality. However, that meta-analysis included only nine studies (seven RCTs and two cohort studies), did not evaluate the risk of bias of included studies, nor did it report data on kidney outcomes.

Provided the findings in this study are genuine, the observed cardiorenal risk benefits of combination therapy of SGLT2 inhibitors and GLP-1 RAs compared with monotherapy may stem from their distinct and potentially synergistic mechanisms of action. SGLT2 inhibitors inhibit glucose and sodium reabsorption in the proximal renal tubule, promoting glucosuria and macula densa activation, resulting in a reduction in glomerular pressure and glomerular hyperfiltration [46, 47]. Additional beneficial cardiometabolic effects may include the promotion of ketogenesis and possibly anti-inflammatory effects [48]. GLP-1 RAs reduce glucagon secretion, delay gastric emptying and result in weight loss. Important cardiorenal protective effects may include a reduction in the rate of atherosclerotic plaque formation and stabilisation of existing plaques due to a reduction in inflammation and fibrosis [49]. The complementary effects of SGLT2 inhibitors and GLP-1 RAs on the development and progression of CKD and heart failure could further enhance the cardiovascular benefits. Recent studies have highlighted the importance of the effects of these agents on adipose tissue (reduction of the rate of atherosclerosis and the development of CKD and heart failure to improve cardiorenal health) [50, 51].

Current guidelines [10, 12] recommend combining SGLT2 inhibitors and GLP-1 RAs to further reduce cardiorenal risk in individuals with type 2 diabetes and established CVD or multiple cardiovascular risk factors. These recommendations are mainly based on earlier studies that have found that combination therapy, compared with monotherapy, is associated with improved control of cardiovascular risk factors, including a greater reduction of HbA_1c_, BP and body weight [16–18]. Our findings provide some support for these recommendations by suggesting that combination therapy with an SGLT2 inhibitor and a GLP-1 RA may further reduce cardiorenal risk in individuals with type 2 diabetes in real-world practice without a clear increase in the risk of specific adverse events such as hypoglycaemia, ketoacidosis and genitourinary tract infections. However, the relatively low number of studies reporting these outcomes, along with differential risks of bias in observational studies with likely unmeasured confounding, precluded drawing robust conclusions.

This systematic review has several limitations. Most importantly, most of the included cohort studies had significant limitations in adjustment for confounding and handling of missing data (ESM Table 6). As with all observational studies, the possibility of residual confounding is inherent to the study design. Additionally, the subpopulations and number of events reported in individuals receiving combination therapy were much lower than in those receiving monotherapy with an SGLT2 inhibitor or a GLP-1 RA. Consequently, the statistical power to detect differences between the groups was relatively low. Although considerable statistical heterogeneity (i.e. *I*^2^ 75–100%) was observed for some of the pooled analyses, the true heterogeneity may be overestimated. This could be attributed to the limited number of studies included in some analyses and the disproportionate influence of larger studies with narrow CIs [52, 53]. Despite these considerations, the populations, interventions and outcomes across the included studies were sufficiently homogeneous to justify pooling. The kidney composite endpoint largely comprised surrogate endpoints such as eGFR decline or albuminuria. Furthermore, we could only make between-study comparisons in the subgroup analyses to assess heterogeneity in treatment effects. It was therefore not possible to distinguish between heterogeneity in results due to heterogeneity between studies or heterogeneity between subgroups. Due to insufficient data, we were unable to evaluate sex-specific differences in the effectiveness of combination therapy or to assess the comparative effectiveness of individual SGLT2 inhibitors or GLP-1 RAs.

### Conclusion

This meta-analysis of observational studies suggests that combining SGLT2 inhibitors and GLP-1 RAs in individuals with type 2 diabetes may lower the risk of MACE, all-cause and cardiovascular mortality, adverse kidney outcomes, hospitalisation for heart failure, stroke and myocardial infarction. These findings highlight potential cardiorenal benefits of combination therapy over monotherapy for individuals with type 2 diabetes in a real-world setting. These results, allied to recent meta-analyses from limited clinical trial data, underscore the need for a dedicated cardiorenal outcome trial to obtain direct evidence for the (cardiorenal) benefits and risks of SGLT2 inhibitor and GLP-1 RA combination therapy.

## Supplementary Information

Below is the link to the electronic supplementary material.

ESM (PDF 3212 KB)

ESM Dataset (XLSX 18 KB)

ESM Syntax (R 137 KB)

## Data Availability

The dataset is available in the ESM Dataset file.

## Abbreviations

aOR: Adjusted OR
CKD: Chronic kidney disease
DPP-4: Dipeptidyl peptidase-4
GLP-1 RA: Glucagon-like peptide-1 receptor agonist
GRADE: Grading of Recommendations Assessment, Development and Evaluation
MACE: Major adverse cardiovascular events
PRISMA: Preferred Reporting Items for Systematic Reviews and Meta-Analyses
SGLT2: Sodium–glucose cotransporter 2

## Code availability

Code/syntax is available in the ESM Syntax file.

## Authors’ relationships and activities

DvR reports receiving personal fees from AstraZeneca, Bayer, Boehringer Ingelheim, Eli Lilly, Novo Nordisk and MSD, outside this manuscript. NS reported receiving personal fees from Abbott Laboratories, AbbVie, Amgen, AstraZeneca, Boehringer Ingelheim, Carmot Therapeutics, Eli Lilly, GlaxoSmithKline, Hanmi Pharmaceuticals, Menarini-Ricerche, Metsera, Novartis, Novo Nordisk, Pfizer and Roche, and research grants from AstraZeneca, Boehringer Ingelheim, Novartis and Roche, outside this manuscript. The authors declare that there are no other relationships or activities that might bias, or be perceived to bias, their work.
