# Supplementary material for: Effectiveness and safety of combining SGLT2 inhibitors and GLP-1 receptor agonists in individuals with type 2 diabetes: a systematic review and meta-analysis of cohort studies
Source: Diabetologia. 2025 Oct 21;69(1):36–49. doi: 10.1007/s00125-025-06565-6 (PMC12686040; doi:10.1007/s00125-025-06565-6)

Supplemental tables and figures

ESM Table 1: Medline search strategy

#1	"diabetes mellitus, type 2"[MeSH Terms] OR "type 2 diabetes"[Title/Abstract] OR "diabetes type 2"[Title/Abstract] OR "T2DM"[Title/Abstract] OR "T2D"[Title/Abstract] OR "DM2"[Title/Abstract] OR "diabetes mellitus type 2"[Title/Abstract]	262,308
#2	"sodium-glucose transporter 2 inhibitors"[MeSH Terms] OR "sodium glucose transporter 2 inhibitor*"[Title/Abstract] OR "sglt 2*"[Title/Abstract] OR "sglt2*"[Title/Abstract] OR "empagliflozin"[Title/Abstract] OR "dapagliflozin"[Title/Abstract] OR "canagliflozin"[Title/Abstract] OR "ertugliflozin"[Title/Abstract]	16,089
#3	"glucagon-like peptide-1 receptor agonists"[MeSH Terms] OR "glucagon like peptide 1 receptor agonist*"[Title/Abstract] OR "glp 1 receptor agonist*"[Title/Abstract] OR "GLP-1 RA"[Title/Abstract] OR "semaglutide"[Title/Abstract] OR "dulaglutide"[Title/Abstract] OR "liraglutide"[Title/Abstract] OR "lixisenatide"[Title/Abstract]	13,643
#4	#1 AND #2 AND #3	1,870
#5	#4 NOT "animals"[MeSH Terms:noexp]	1,792
#6	#5 NOT ("review"[Publication Type] OR "systematic review"[Publication Type] OR "meta analysis"[Publication Type])	971

ESM Table 2: Embase search strategy

#1	'non insulin dependent diabetes mellitus'/exp OR 'type 2 diabetes':ab,ti,kw OR 'diabetes type 2':ab,ti,kw OR 'T2DM':ab,ti,kw OR 'T2D':ab,ti,kw OR 'DM2':ab,ti,kw	462,305
#2	'sodium glucose cotransporter 2 inhibitor'/exp OR 'sodium-glucose cotransporter 2 inhibitor*':ab,ti,kw OR 'sglt-2*':ab,ti,kw OR 'sglt2*':ab,ti,kw OR 'empagliflozin':ab,ti,kw OR 'dapagliflozin':ab,ti,kw OR 'canagliflozin':ab,ti,kw OR 'ertugliflozin':ab,ti,kw	39,907
#3	'glucagon like peptide-1 receptor agonists'/exp OR 'glucagon-like peptide-1 receptor agonist*':ab,ti,kw OR 'GLP-1 receptor agonist*':ab,ti,kw OR 'GLP-1 RA':ab,ti,kw OR 'semaglutide':ab,ti,kw OR 'dulaglutide':ab,ti,kw OR 'liraglutide':ab,ti,kw OR 'lixisenatide':ab,ti,kw	61,279
#4	#1 AND #2 AND #3	8,302
#5	#4 NOT ('nonhuman'/de)	7,455
#6	#5 NOT ('conference abstract'/it OR 'review'/it OR 'meta analysis'/de OR 'systematic review'/de)	4,223

ESM Table 3: List of excluded studies during full-text screening and reasons for exclusion

**Reason for exclusion**	***N***	**References**
No outcome of interest reported	24	- Lunati ME, Cimino V, Bernasconi D, Gandolfi A, Morpurgo PS, Tinari C, et al. Type 2 diabetes mellitus pharmacological remission with dapagliflozin plus oral semaglutide. Pharmacol Res. 2024;199:107040.<br>- Bechlioulis A, Markozannes G, Chionidi I, Liberopoulos E, Naka KK, Ntzani EE, et al. The effect of SGLT2 inhibitors, GLP1 agonists, and their sequential combination on cardiometabolic parameters: A randomized, prospective, intervention study. J Diabetes Complications. 2023;37(4):108436.<br>- Katogiannis K, Thymis J, Kousathana F, Pavlidis G, Korakas E, Kountouri A, et al. Effects of liraglutide, empagliflozin and their combination on left atrial strain and arterial function. Medicina (Kaunas). 2024;60(3):395.<br>- Gullaksen S, Vernstrøm L, Sørensen SS, Funck KL, Petersen L, Bek T, et al. Effects of semaglutide and empagliflozin on oxygenation, vascular

autoregulation, and central thickness of the retina in people with type 2 diabetes: A prespecified secondary analysis of a randomised clinical trial. J Diabetes Complications. 2023;37(5):108472.

- Ikonomidis I, Pavlidis G, Thymis J, Birba D, Kalogeris A, Kousathana F, et al. Effects of glucagon-like peptide-1 receptor agonists, sodium-glucose cotransporter-2 inhibitors, and their combination on endothelial glycocalyx, arterial function, and myocardial work index in patients with type 2 diabetes mellitus after 12-month treatment. J Am Heart Assoc. 2020;9(9):e015716.
- Gullaksen S, Vernstrøm L, Sørensen SS, Ringgaard S, Laustsen C, Funck KL, et al. Separate and combined effects of semaglutide and empagliflozin on kidney oxygenation and perfusion in people with type 2 diabetes: A randomised trial. Diabetologia. 2023;66(5):813-25.
- Gullaksen S, Vernstrøm L, Sørensen SS, Ringgaard S, Laustsen C, Funck KL, Poulsen PL, Laugesen E. Correction: Separate and combined effects of semaglutide and empagliflozin on kidney oxygenation and perfusion in people with type 2 diabetes: a randomised trial. Diabetologia. 2024;67(7):1451.
- Ou T, Wang W, Yong H, Hao H, Wang R, Dai X, et al. Liraglutide plus dapagliflozin for high uric acid and microalbuminuria in diabetes mellitus complicated with metabolic syndrome. Altern Ther Health Med. 2022;28(6):14-21.
- Blanco CA, Garcia K, Singson A, Smith WR. Use of SGLT2 inhibitors reduces heart failure and hospitalization: A multicenter, real-world evidence study. Perm J. 2023;27(1):77-87.
- Kobayashi K, Toyoda M, Hatori N, Tsukamoto S, Kimura M, Sakai H, et al. The concomitant use of sodium-glucose co-transporter 2 inhibitors improved the renal outcome of Japanese patients with type 2 diabetes treated with glucagon-like peptide 1 receptor agonists. Cardiovasc Endocrinol Metab. 2023;12(4):e0292.
- Feher M, Hinton W, Forbes A, Munro N, Joy M, Wheeler D, et al. Sodium-glucose cotransporter-2 inhibitor and glucagon-like peptide-1 receptor agonist combination therapy in type 2 diabetes: Protocol for a kidney end points real-world study (COMBi-KID Study). JMIR Res Protoc. 2022;11(7):e34206.
- Lambadiari V, Thymis J, Kouretas D, Skaperda Z, Tekos F, Kousathana F, et al. Effects of a 12-month treatment with glucagon-like peptide-1 receptor agonists, sodium-glucose cotransporter-2 inhibitors, and their combination on oxidant and antioxidant biomarkers in patients with type 2 diabetes. Antioxidants (Basel). 2021;10(9):1379.
- Ou T, Wang W, Yong H, Hao H, Wang R, Dai X, et al. Liraglutide plus dapagliflozin for high uric acid and microalbuminuria in diabetes mellitus complicated with metabolic syndrome. Altern Ther Health Med. 2022;28(6):14-21.
- Kobayashi K, Toyoda M, Hatori N, Sato K, Miyakawa M, Tamura K, et al. The comparison of the kidney effects of dipeptidyl peptidase 4 inhibitors and glucagon-like peptide 1 agonist-administered concomitant with sodium-glucose cotransporter 2 inhibitors in Japanese patients with type 2 diabetes mellitus and chronic kidney disease. J Diabetes Res. 2021;2021:6573369.
- Gastaldelli A, Repetto E, Guja C, Hardy E, Han J, Jabbour SA, et al. Exenatide and dapagliflozin combination improves markers of liver steatosis and fibrosis in patients with type 2 diabetes. Diabetes Obes Metab. 2020;22(3):393-403.
- Ferrannini E, Baldi S, Frías JP, Guja C, Hardy E, Repetto E, et al. Hormone-substrate changes with exenatide plus dapagliflozin versus each drug alone: The randomized, active-controlled DURATION-8 study. Diabetes Obes Metab. 2020;22(1):99-106.
- Berkovic MC, Bilic-Curcic I, Bozek T, Mahecic DH, Majanovic SK, Canecki-Varzic S, et al. Glucagon-like-1 receptor agonists and sodium/glucose cotransporter-2 inhibitors combination-are we exploiting their full potential in a real life setting? World J Diabetes. 2020;11(11):540-52.
- Carretero Gómez J, Arévalo Lorido JC, Gómez Huelgas R, García de Lucas D, Mateos Polo L, Varela Aguilar JM, et al. Combination therapy with glucagon-like peptide-1 receptor agonists and sodium-glucose cotransporter 2

		inhibitors in older patients with type 2 diabetes: A real-world evidence study. Can J Diabetes. 2019;43(3):186-92.<br>- Jabbour SA, Frías JP, Guja C, Hardy E, Ahmed A, Öhman P, et al. Effects of exenatide once weekly plus dapagliflozin, exenatide once weekly, or dapagliflozin, added to metformin monotherapy, on body weight, systolic blood pressure, and triglycerides in patients with type 2 diabetes in the DURATION-8 study. Diabetes Obes Metab. 2018;20(6):1515-9.<br>- Frías JP, Hardy E, Ahmed A, Öhman P, Jabbour S, Wang H, et al. Effects of exenatide once weekly plus dapagliflozin, exenatide once weekly alone, or dapagliflozin alone added to metformin monotherapy in subgroups of patients with type 2 diabetes in the DURATION-8 randomized controlled trial. Diabetes Obes Metab. 2018;20(6):1520-5.<br>- Lin YH, Zhang ZJ, Zhong JQ, Wang ZY, Peng YT, Lin YM, et al. Semaglutide combined with empagliflozin vs. monotherapy for non-alcoholic fatty liver disease in type 2 diabetes: Study protocol for a randomized clinical trial. PLoS One. 2024;19(5):e0302155.<br>- Brown E, Wilton MM, Sprung VS, Harrold JA, Halford JCG, Stancak A, et al. A randomised, controlled, double blind study to assess mechanistic effects of combination therapy of dapagliflozin with exenatide QW versus dapagliflozin alone in obese patients with type 2 diabetes mellitus (RESILIENT): study protocol. BMJ Open. 2021;11(7):e045663.<br>- Berra C, Manfrini R, Bifari F, Cipponeri E, Ghelardi R, Centofanti L, et al. Improved glycemic and weight control with dulaglutide addition in SGLT2 inhibitor treated obese type 2 diabetic patients at high cardiovascular risk in a real-world setting: The AWARE-2 study. Pharmacol Res. 2024;210:107517.<br>- Nigam A. Improved clinical outcomes with the combination therapy of a glucagon-like peptide-1 receptor agonist and a sodium-glucose cotransporter-2 inhibitor in overweight/obese people with type 2 diabetes: Real-world evidence from the Indian subcontinent. J Assoc Physicians India. 2024;72(9):37-42.
No comparison of SGLT2 inhibitor and GLP-1 RA combination therapy with monotherapy	19	- Trombara F, Cosentino N, Bonomi A, Ludergnani M, Poggio P, Gionti L, et al. Impact of chronic GLP-1 RA and SGLT-2I therapy on in-hospital outcome of diabetic patients with acute myocardial infarction. Cardiovasc Diabetol. 2023;22(1):26.<br>- Kobayashi K, Toyoda M, Tone A, Kawanami D, Suzuki D, Tsuriya D, et al. Renoprotective effects of combination treatment with sodium-glucose cotransporter inhibitors and GLP-1 receptor agonists in patients with type 2 diabetes mellitus according to preceding medication. Diab Vasc Dis Res. 2023;20(6):14791641231222837.<br>- Ciardullo S, Savaré L, Rea F, Perseghin G, Corrao G. Adherence to GLP1-RA and SGLT2-I affects clinical outcomes and costs in patients with type 2 diabetes. Diabetes Metab Res Rev. 2024;40(4):e3791.<br>- Kaku K, Nakayama Y, Yabuuchi J, Naito Y, Kanasaki K. Safety and effectiveness of empagliflozin in clinical practice as monotherapy or with other glucose-lowering drugs in Japanese patients with type 2 diabetes: Subgroup analysis of a 3-year post-marketing surveillance study. Expert Opin Drug Saf. 2023;22(9):819-32.<br>- Kim HS, Lee WJ. Clinical efficacy of sodium-glucose cotransporter 2 inhibitor and glucagon-like peptide-1 receptor agonist combination therapy in type 2 diabetes mellitus: Real-world study. Diabetes Metab J. 2022;46(4):665-6.<br>- Guo L, Li L, Yu Q, Wang N, Chen J, Wang Z, et al. Study design and baseline characteristics of patients with T2DM in the post-marketing safety study of dulaglutide in China (TRUST-CHN). Diabetes Ther. 2022;13(6):1231-44.<br>- Wright AK, Carr MJ, Kontopantelis E, Leelarathna L, Thabit H, Emsley R, et al. Primary prevention of cardiovascular and heart failure events with SGLT2 inhibitors, GLP-1 receptor agonists, and their combination in type 2 diabetes. Diabetes Care. 2022;45(4):909-18.<br>- Long-term effects of dapagliflozin plus saxagliptin versus glimepiride on a background of metformin in patients with type 2 diabetes: Results of a 104-week extension to a 52-week randomized, phase 3 study and liver fat MRI substudy. Drugs Real World Outcomes. 2024;11(1):81-90.<br>- Ludvik B, Giorgino F, Jódar E, Frias JP, Fernández Landó L, Brown K, et al. Once-weekly tirzepatide versus once-daily insulin degludec as add-on to

		metformin with or without SGLT2 inhibitors in patients with type 2 diabetes (SURPASS-3): A randomised, open-label, parallel-group, phase 3 trial. Lancet. 2021;398(10300):583-98.<br>- Sethi B, Sahay R, Tiwaskar M, Negalur V, Dhediya R, Gaurav K, et al. Effectiveness of dapagliflozin as add-on to metformin with or without other oral antidiabetic drugs in type 2 diabetes mellitus: A multicentre, retrospective, real-world database study. Drugs Real World Outcomes. 2024;11(1):81-90.<br>- Terauchi Y, Utsunomiya K, Yasui A, Seki T, Cheng G, Shiki K, et al. Safety and efficacy of empagliflozin as add-on therapy to GLP-1 receptor agonist (liraglutide) in Japanese patients with type 2 diabetes mellitus: A randomised, double-blind, parallel-group phase 4 study. Diabetes Ther. 2019;10(3):951-63.<br>- Kim KS. Letter: Predictors of the therapeutic efficacy and consideration of the best combination therapy of sodium-glucose cotransporter 2 inhibitors. Diabetes Metab J. 2019;43(3):377-8.<br>- Ghosal S, Sinha B. Liraglutide and dulaglutide therapy in addition to SGLT-2 inhibitor and metformin treatment in Indian type 2 diabetics: A real world retrospective observational study. Clin Diabetes Endocrinol. 2018;4:11.<br>- Kaku K, Maegawa H, Tanizawa Y, Kiyosue A, Ide Y, Tokudome T, et al. Dapagliflozin as monotherapy or combination therapy in Japanese patients with type 2 diabetes: An open-label study. Diabetes Ther. 2014;5(2):415-33.<br>- Ciardullo S, Savaré L, Rea F, Perseghin G, Corrao G. Adherence to GLP1-RA and SGLT2-I affects clinical outcomes and costs in patients with type 2 diabetes. Diabetes Metab Res Rev. 2024;40(4):e3791.<br>- RECAP Study Group, Tsukamoto S, Kobayashi K, Toyoda M, Tone A, Kawanami D, Suzuki D, et al. Effect of preceding drug therapy on the renal and cardiovascular outcomes of combined sodium-glucose cotransporter-2 inhibitor and glucagon-like peptide-1 receptor agonist treatment in patients with type 2 diabetes and chronic kidney disease. Diabetes Obes Metab. 2024;26(8):3248-60.<br>- Diker Cohen T, Polansky A, Bergman I, Ayada G, Babich T, Akirov A, et al. Safety of sodium-glucose cotransporter 2 inhibitors in kidney transplant recipients with diabetes mellitus. Diabetes Metab. 2025;51(3):101627.<br>- RECAP study group, Tsukamoto S, Kobayashi K, Toyoda M, Tone A, Kawanami D, et al. Effect of preceding drug therapy on the renal and cardiovascular outcomes of combined sodium-glucose cotransporter-2 inhibitor and glucagon-like peptide-1 receptor agonist treatment in patients with type 2 diabetes and chronic kidney disease. Diabetes Obes Metab. 2024;26(8):3248-60.<br>- Naoum I, Saliba W, Barnett-Griness O, Aker A, Zafrir B. Glucose-lowering drugs with proven cardiovascular benefit following acute coronary syndrome in patients with type 2 diabetes: Treatment gaps and outcomes. J Clin Med. 2024;13(18):5541.
Not a cohort study	53	- Packer M. Should we be combining GLP-1 receptor agonists and SGLT2 inhibitors in treating diabetes? Am J Med. 2018;131(5):461-3.<br>- Díaz-Trastoy O, Villar-Taibo R, Sifontes-Dubón M, Mozo-Peñalver H, Bernabeu-Morón I, Cabezas-Agrícola JM, et al. GLP1 receptor agonist and SGLT2 inhibitor combination: An effective approach in real-world clinical practice. Clin Ther. 2020;42(2):e1-12.<br>- McCulley L, Hurren KM. Safety and efficacy of GLP-1 receptor agonists and SGLT2 inhibitors among veterans with type 2 diabetes. Fed Pract. 2022;39(Suppl 5):e0319.<br>- Kim HS, Yoon T, Jung CH, Park JY, Lee WJ. Clinical efficacy of sodium-glucose cotransporter 2 inhibitor and glucagon-like peptide-1 receptor agonist combination therapy in type 2 diabetes mellitus: Real-world study. Diabetes Metab J. 2022;46(4):658-62.<br>- Busch RS, Kane MP. Combination SGLT2 inhibitor and GLP-1 receptor agonist therapy: A complementary approach to the treatment of type 2 diabetes. Postgrad Med. 2017;129(7):686-97.<br>- Schernthaner-Reiter MH, Schernthaner G. Combination therapy of SGLT2 inhibitors with incretin-based therapies for the treatment of type 2 diabetes

mellitus: Effects and mechanisms of action. Expert Rev Endocrinol Metab. 2016;11(3):281-96.
- Patoulias D, Michailidis T. SGLT-2 inhibitor and GLP-1 receptor agonist treatment for patients with nonalcoholic fatty liver disease and type 2 diabetes mellitus: Is their combination the optimal treatment option? J Clin Transl Hepatol. 2022;10(4):574-6.
- Ebell MH. For patients with type 2 diabetes mellitus who are taking a GLP-1 receptor agonist, an SGLT2 inhibitor may be preferred to a sulfonylurea as add-on therapy. Am Fam Physician. 2021;104(4):424.
- Kuhadiya ND, Mahmood I. Effects of concomitant combination of SGLT-2 inhibitor and GLP-1 receptor agonist on renal outcomes in T2D with eGFR below 30 and macroalbuminuria: A case series. Clin Case Rep. 2021;9(4):2310-6.
- Tanner M. In type 2 diabetes, GLP-1 RA plus SGLT2 inhibitor vs. either drug alone reduces HbA1c and SBP and may reduce body weight. Ann Intern Med. 2020;173(10):JC52.
- Terauchi Y, Fujiwara H, Kurihara Y, Suganami H, Tamura M, Senda M, et al. Long-term safety and efficacy of the sodium-glucose cotransporter 2 inhibitor, tofogliflozin, added on glucagon-like peptide-1 receptor agonist in Japanese patients with type 2 diabetes mellitus: A 52-week open-label, multicenter, post-marketing clinical study. J Diabetes Investig. 2019;10(6):1518-26.
- Ishihara H, Yamaguchi S, Nakao I, Sakatani T. Ipragliflozin add-on therapy to a GLP-1 receptor agonist in Japanese patients with type 2 diabetes (AGATE): A 52-week open-label study. Diabetes Ther. 2018;9(4):1549-67.
- Harashima SI, Inagaki N, Kondo K, Maruyama N, Otsuka M, Kawaguchi Y, et al. Efficacy and safety of canagliflozin as add-on therapy to a glucagon-like peptide-1 receptor agonist in Japanese patients with type 2 diabetes mellitus: A 52-week, open-label, phase IV study. Diabetes Obes Metab. 2018;20(7):1770-5.
- Seino Y, Yabe D, Sasaki T, Fukatsu A, Imazeki H, Ochiai H, et al. Sodium-glucose cotransporter-2 inhibitor luseogliflozin added to glucagon-like peptide-1 receptor agonist liraglutide improves glycemic control with bodyweight and fat mass reductions in Japanese patients with type 2 diabetes: A 52-week, open-label, single-arm study. J Diabetes Investig. 2018;9(2):332-40.
- Siegmund-Schultze N. Combination therapy in type 2 diabetes: Exenatide plus dapagliflozin: Efficacy has increased. Deutsches Arzteblatt Int. 2017;114:7.
- Pratley RE, Cersosimo E. Use of canagliflozin in combination with and compared to incretin-based therapies in type 2 diabetes. Clin Diabetes. 2017;35(3):141-53.
- Nauck MA, Meier JJ. GLP-1 receptor agonists and SGLT2 inhibitors: A couple at last? Lancet Diabetes Endocrinol. 2016;4(12):963-4.
- Curtis L, Humayun MA, Walker J, Hampton K, Partridge H. Addition of SGLT2 inhibitor to GLP-1 agonist therapy in people with type 2 diabetes and suboptimal glycaemic control. Pract Diabetes. 2016;33(4):129-32.
- Bloomgarden ZT, Handelsman Y. SGLT-2 inhibition added to GLP-1 agonist therapy for type 2 diabetes: What is the benefit? Endocr Pract. 2015;21(12):1442-4.
- Saroka RM, Kane MP, Busch RS, Watsky J, Hamilton RA. SGLT-2 inhibitor therapy added to GLP-1 agonist therapy in the management of T2DM. Endocr Pract. 2015;21(12):1315-22.
- Jayasinghe KNU, Greener VJ, Feher MD. Combining SGLT2 inhibitor and GLP-1 agonist: Exaggerated weight loss in a morbidly obese patient with type 2 diabetes. Br J Diabetes Vasc Dis. 2016;16(3):138-9.
- Ohki T, Isogawa A, Toda N, Tagawa K. Effectiveness of ipragliflozin, a sodium-glucose co-transporter 2 inhibitor, as a second-line treatment for non-alcoholic fatty liver disease patients with type 2 diabetes mellitus who do not respond to incretin-based therapies. Clin Drug Investig. 2016;36(4):313-9.
- de Oliveira Costa J, Lin J, Milder TY, Greenfield JR, Day RO, Stocker SL, et al. Geographic variation in sodium-glucose cotransporter 2 inhibitor and glucagon-like peptide-1 receptor agonist use in people with type 2 diabetes in New South Wales, Australia. Diabetes Obes Metab. 2024;26(7):2787-95.

		- Ferreira JP, Mendonça L, Neves JS. Does semaglutide reduce kidney disease events on top of SGLT2 inhibitors in patients with CKD and T2D? J Card Fail. 2024;30(9):1181-2.<br>- Limonte CP, Hall YN, Trikudanathan S, Tuttle KR, Hirsch IB, de Boer IH, et al. Prevalence of SGLT2i and GLP1RA use among US adults with type 2 diabetes. J Diabetes Complications. 2022;36(6):108204.<br>- Lam CSP, Ramasundarahettige C, Branch KRH, Sattar N, Rosenstock J, Pratley R, et al. Efpeglenatide and clinical outcomes with and without concomitant sodium-glucose cotransporter-2 inhibition use in type 2 diabetes: Exploratory analysis of the AMPLITUDE-O trial. Circulation. 2022;145(8):565-74.<br>- Arnott C, Neuen BL, Heerspink HJL, Figtree GA, Kosiborod M, Lam CS, et al. The effects of combination canagliflozin and glucagon-like peptide-1 receptor agonist therapy on intermediate markers of cardiovascular risk in the CANVAS program. Int J Cardiol. 2020;318:126-9.<br>- Arnott C, Li J-W, Cannon CP, de Zeeuw D, Neuen BL, Heerspink HJL, et al. The effects of canagliflozin on heart failure and cardiovascular death by baseline participant characteristics: Analysis of the CREDENCE trial. Diabetes Obes Metab. 2021;23(7):1652-9.<br>- Heerspink HJL, Stefánsson BV, Correa-Rotter R, Chertow GM, Greene T, Hou F-F, et al. Dapagliflozin in patients with chronic kidney disease. N Engl J Med. 2020;383(15):1436-46.<br>- McMurray JV, Solomon SD, Inzucchi SE, Køber L, Kosiborod MN, Martinez FA, et al. Dapagliflozin in patients with heart failure and reduced ejection fraction. N Engl J Med. 2019;381(21):1995-2008.<br>- Cahn A, Wiviott SD, Mosenzon O, Murphy SA, Goodrich EL, Yanuv I, et al. Cardiorenal outcomes with dapagliflozin by baseline glucose-lowering agents: Post hoc analyses from DECLARE-TIMI 58. Diabetes Obes Metab. 2021;23(1):29-38.<br>- Solomon SD, McMurray JV, Claggett B, de Boer RA, DeMets D, Hernandez AF, et al. Dapagliflozin in heart failure with mildly reduced or preserved ejection fraction. N Engl J Med. 2022;387(12):1089-98.<br>- Jabbour SA, Frías JP, Ahmed A, Hardy E, Choi J, Sjöström CD, Guja C. Efficacy and safety over 2 years of exenatide plus dapagliflozin in the DURATION-8 study: A multicenter, double-blind, phase 3, randomized controlled trial. Diabetes Care. 2020;43(10):2528-36.<br>- The EMPA-KIDNEY collaborative group. Empagliflozin in patients with chronic kidney disease. N Engl J Med. 2023;388(2):117-27.<br>- Zinman B, Wanner C, Lachin JM, Fitchett D, Bluhmki E, Hantel S, et al. Empagliflozin, cardiovascular outcomes, and mortality in type 2 diabetes. N Engl J Med. 2015;373(22):2117-28.<br>- Anker SD, Butler J, Filippatos G, Ferreira JP, Bocchi E, Böhm M, et al. Empagliflozin in heart failure with a preserved ejection fraction. N Engl J Med. 2021;385(16):1451-61.<br>- Packer M, Anker SD, Butler J, Filippatos G, Pocock SJ, Carson P, et al. Cardiovascular and renal outcomes with empagliflozin in heart failure. N Engl J Med. 2020;383(15):1413-24.<br>- Clegg LE, Penland RC, Bachina S, Boulton DW, Thuresson M, Heerspink HJL, et al. Effects of exenatide and open-label SGLT2 inhibitor treatment, given in parallel or sequentially, on mortality and cardiovascular and renal outcomes in type 2 diabetes: Insights from the EXSCEL trial. Cardiovasc Diabetol. 2019;18(1):138.<br>- Mann JFE, Rossing P, Bakris G, Belmar N, Bosch-Traberg H, Busch R, et al. Effects of semaglutide with and without concomitant SGLT2 inhibitor use in participants with type 2 diabetes and chronic kidney disease in the FLOW trial. Nat Med. 2024;30(10):2849-56.<br>- Neves JS, Borges-Canha M, Vasques-Nóvoa F, Green JB, Leiter LA, Granger CB, et al. GLP-1 receptor agonist therapy with and without SGLT2 inhibitors in patients with type 2 diabetes. J Am Coll Cardiol. 2023;82(6):517-25.<br>- Bhatt DL, Szarek M, Pitt B, Cannon CP, Leiter LA, McGuire DK, et al. Sotagliflozin in patients with diabetes and chronic kidney disease. N Engl J Med. 2021;384(2):129-39.<br>- McGuire DK, Busui RP, Deanfield J, Inzucchi SE, Mann JFE, Marx N, et al. Effects of oral semaglutide on cardiovascular outcomes in individuals with type 2 diabetes and established atherosclerotic cardiovascular disease

		and/or chronic kidney disease: Design and baseline characteristics of SOUL, a randomized trial. Diabetes Obes Metab. 2023;25(7):1932-41.<br>- Cannon CP, Pratley R, Dagogo-Jack S, Mancuso J, Huyck S, Masiukiewicz U, et al. Cardiovascular outcomes with ertugliflozin in type 2 diabetes. N Engl J Med. 2020;383(15):1425-35.<br>- Wang J, Chen Y, Lu Y, Wang H, Zhao Y. Efficacy and safety of glucagon-like peptide-1 receptor agonist combined with sodium-glucose co-transporter-2 inhibitor in the treatment of type 2 diabetes mellitus patients with obesity: A retrospective analysis study. Am J Transl Res. 2023;15(4):2949-56.<br>- Ferreira JP, Mendonça L, Neves JS. Does semaglutide reduce kidney disease events on top of SGLT2 inhibitors in patients with CKD and T2D? J Card Fail. 2024;30(9):1181-2.<br>- Ikizler TA. The combination of SGLT2 inhibitors and glucagon-like peptide-1 receptor agonists: Are 2 drugs better than 1? Kidney Int. 2025;107(3):385-8.<br>- Osman AA, Tayeb BA, Metzendorf MI, Bongaerts B, Mohammed N, Njangiru IK, et al. Glucagon-like peptide-1 receptor agonists and sodium-glucose co-transporter-2 inhibitors combination therapy for adults with type 2 diabetes mellitus: A network meta-analysis. Cochrane Database Syst Rev. 2025;4(4):CD015952.<br>- Qasim R, Moin F, Kamran. Effect of combination treatment with GLP-1 receptor agonist and SGLT-2 inhibitors on incidence of cardiovascular and serious renal events. Med Forum Mon. 2024;35(6):59-63.<br>- Scheen AJ. GLP-1 receptor agonists and SGLT2 inhibitors in type 2 diabetes: Pleiotropic cardiometabolic effects and add-on value of a combined therapy. Drugs. 2024;84(11):1347-64.<br>- Tang M, Morieri ML, Kalim S, Doria A. Combination therapy with SGLT2 inhibitors and GLP-1 receptor agonists for diabetic kidney disease. J Am Soc Nephrol. 2025;36(4):726-9.<br>- Wu Y, Yang Z, Cao Q. Efficacy and safety of GLP-1 receptor agonists combined with SGLT-2 inhibitors in elderly patients with type 2 diabetes: A meta-analysis. Am J Transl Res. 2024;16(11):6852-66.<br>- Zhang M, Lin C, Cai X, Jiao R, Bai S, Li Z, et al. One or two? Comparison of the cardiorenal effects between combination therapy and monotherapy with SGLT2i or GLP1RA. Diabetes Obes Metab. 2025;27(2):806-15.<br>- Marx N, Deanfield JE, Mann JFE, Arechavaleta R, Bain SC, Bajaj HS, et al; SOUL study group. Oral semaglutide and cardiovascular outcomes in persons with type 2 diabetes, according to SGLT2i use: Prespecified analyses of the SOUL randomized trial. Circulation. 2025. doi: 10.1161/CIRCULATIONAHA.125.074545.
Maximum follow-up < 12 months	5	- Vernstrøm L, Gullaksen S, Sørensen SS, Funck KL, Laugesen E, Poulsen PL. Separate and combined effects of empagliflozin and semaglutide on vascular function: A 32-week randomized trial. Diabetes Obes Metab. 2024;26(5):1624-35.<br>- Brown E, Wilton MM, Sprung VS, Harrold JA, Halford JCG, Stancak A, et al. A randomised, controlled, double-blind study to assess mechanistic effects of combination therapy of dapagliflozin with exenatide QW versus dapagliflozin alone in obese patients with type 2 diabetes mellitus (RESILIENT): Study protocol. BMJ Open. 2021;11(7):e045663.<br>- Li Q, Wang X, Guo A, Zheng W, Bi J, He Y, et al. The promising significance of liraglutide combined with dapagliflozin or empagliflozin in the prevention of early diabetic nephropathy. Am J Transl Res. 2022;14(8):5622-9.<br>- Zinman B, Bhosekar V, Busch R, Holst I, Ludvik B, Thielke D, et al. Semaglutide once weekly as add-on to SGLT-2 inhibitor therapy in type 2 diabetes (SUSTAIN 9): A randomised, placebo-controlled trial. Lancet Diabetes Endocrinol. 2019;7(5):356-67.<br>- Guja C, Giorgino F, Blonde L, Ali A, Prázný M, Meier JJ, et al. Concomitant iGlarLixi and sodium-glucose co-transporter-2 inhibitor therapy in adults with type 2 diabetes: LixiLan-G trial and real-world evidence results. Diabetes Ther. 2022;13(1):205-15.

ESM Table 4: Baseline medication use of included cohort studies

Study (publication year)	RAAS-inhibitors	Other anti-hypertensives	Metformin	Sulphonylurea	Insulin	DPP-4 inhibitor	Other glucose-lowering drugs	Antiplatelets	Lipid-lowering drugs
Chaiyakunapruk et al (2025) [29]	ACE-i: 71,408 (43)<br>ARB: 65,467 (40)	Betablocker: 94,645 (57)<br>Loop diuretics: 35,497 (22)<br>MRA: 16,307 (10)<br>Other diuretics: 45,341 (28)	117,573 (71)	46,263 (28)	38,685 (23)	44,120 (27)	12,733 (8)	32,628 (20)	146,777 (89)
Dave et al (2021) [30]	10,580 (42)	Betablocker: 79,70 (32)<br>Diuretics: 2597 (10)	17,722 (70)	-	6381 (25)	-	-	1870 (7)	17,035 (68)
García-Vega et al (2024) [25]	-	-	-	-	-	-	-	-	-
Gorgojo-Martínez et al (2017) [31]	157 (74)	6 (3)	192 (90)	35 (16)	90 (42)	43 (20)	8 (4)	-	176 (83)
Horiuchi et al 2025 [26]	-	-	-	-	-	-	-	-	-
Jensen et al (2020) [32]	-	Anti-hypertensives: 9129 (55)	16,664 (100)	-	4921 (30)	-	-	9129 (55)	14,076 (84)
Jhu et al (2024) [33]	ACE-i: 61,177 (43)<br>ARB: 46,205 (32)	Betablocker: 55,399 (39)<br>CCB: 39,825 (28)<br>Diuretic: 55,426 (39))	-	-	72,406 (51)	-	Oral antidiabetics 140,485 (99)	-	114,920 (81)
Kobayashi et al (2023) [34]	219 (59)	Betablocker: 58 (16)<br>CCB: 173 (47)	191 (51)	71 (19)	172 (46)	-	TZD: 60 (16)	-	224 (60)
Lau et al (2022) [35]	1869 (65)	320 (11)	2654 (92)	1326 (46)	1655 (57)	-	240 (8)	835 (29)	2426 (84)
Liu et al (2025) [36]	227 (55)	Betablocker: 247 (59)<br>CCB: 150 (36)	362 (87)	-	235 (56)	-	-	100 (24)	399 (96)
Lopez et al (2022) [37]	677 (99)	Betablocker: 646 (94)<br>MRA: 447 (65)<br>Loop diuretic: 528 (77)	639 (93)	-	504 (74)	-	-	369 (54)	672 (98)
Luo et al (2023) [38]	834 (50)	Betablocker: 343 (21)<br>CCB: 541 (32)<br>Diuretics: 501 (30)	1623 (97)	1069 (64)	1345 (80)	489 (29)	832 (49)	346 (21)	814 (49)
Marfella et al (2024) [39]	ACE-i: 190 (43)<br>ARB: 170 (38)	Betablocker: 228 (52)<br>CCB: 143 (32)	342 (77)	-	164 (37)	38 (9)	43 (10)	279 (63)	272 (62)
Patel et al (2024) [40]	8159 (58)	Betablocker: 11,123 (79)<br>Loop diuretics: 8913 (63)<br>Thiazide: 6336 (45)<br>MRA: 3851 (27)	9401 (67)	3044 (22)	9850 (70)	-	-	9461 (67)	11,800 (84)
Riley et al (2023) [41]	-	-	-	-	311577 (100)	-	-	-	-
Schechter et al (2023) [42]	5129 (75)	5471 (80)	6651 (97)	3718 (54)	69 (1)	-	480 (7)	-	-
Simms-Williams et al (2024a) [43]	ACE-i: 6582 (49)<br>ARB: 27,55 (21)	Betablocker: 3223 (24)<br>CCB: 4265 (32)<br>Diuretic: 2290 (17)<br>Other: 191 (1)	12,083 (90)	6282 (47)	3406 (25)	3400 (25)	TZD: 936 (7)<br>Meglitinides: 47 (0)<br>α-glucosidase inhibitors: 11 (0)	Aspirin: 3353 (25)<br>Other: 772 (6)	Statin: 11,000 (82)<br>Fibrates: 427 (3)
Simms-Williams et al (2024b) [43]	ACE-i: 8828 (49)<br>ARB: 3341 (19)	Betablocker: 4109 (23)<br>CCB: 5390 (30)<br>Diuretic: 2732 (15)<br>Other: 229 (1)	16,143 (90)	7677 (43)	3121 (18)	7654 (43)	TZD: 991 (6)<br>Meglitinides: 72 (0)<br>α-glucosidase inhibitors: 24 (0)	Aspirin: 3809 (21)<br>Other: 1179 (7)	Statin: 14,736 (82)<br>Fibrate: 492 (3)

Wright et al (2022a) [44]	592 (56)	Betablocker: 111 (10)<br>Diuretic: 293 (28)<br>Other: 89 (8)	910 (86)	420 (40)	-		TZD: 233 (22)	179 (17)	712 (67)
Wright et al (2022b) [44]	9612 (62)	Betablocker: 2329 (15)<br>Diuretic 3870 (25)<br>CCB: 4297 (28)<br>Other: 1746 (11)	13,088 (85)	6337 (41)	-	3271 (21)	TZD: 635 (4)	3921 (25)	7684 (50)
Wright et al (2022c) [44]	4335 (63)	Betablocker: 1107 (16)<br>Diuretic 1672 (24)<br>CCB: 1981 (29)<br>Other: 1082 (16)	5747 (84)	2945 (43)	-	1233 (18)	TZD: 285 (4)	2015 (29)	5311 (77)

Abbreviations: ACE-I, ACE inhibitor; ARB, angiotensin receptor blocker; ARNi, angiotensin receptor-neprilysin inhibitor; CCB, calcium channel blocker; DPP-4, dipeptidyl-peptidase-4; P2Y12-I, P2Y12 inhibitor; RAAS-inhibitors, renin-angiotensin-aldosterone system inhibitors; TZD, thiazolidinediones.
* including anticoagulants

ESM Table 5: Key eligibility criteria and reported outcomes of included cohort studies

**Study (publication year)**	**Key inclusion criteria**	**Key exclusion criteria**	**Reported outcomes**	**Definition of outcome**
Chaiyakunapruk et al (2025) [29]	T2D<br>Age ≥18 years<br>Treated with SGLT2i	T1D<br>Pregnancy<br>Missing data for age or sex<br>Initiation of another new glucose-lowering therapy on the index date<br>Prevalent GLP-1 RA user<br>Major adverse cardiovascular event outcome within 60 days before the index date.	MACE	Composite of non-fatal ischemic stroke, non-fatal MI and all-cause mortality
			Non-fatal myocardial infarction	
			Non-fatal stroke	
Dave et al (2021) [30]	T2D	T1D, cancer, end-stage renal disease, HIV<br>Living in a nursing home or hospice care	MACE	Composite of hospitalizations for myocardial infarction or stroke and all-cause mortality
			All-cause mortality	
			Nonfatal myocardial infarction	
			Nonfatal stroke	
			Heart failure hospitalisation	
García-Vega et al (2024) [25]	Prescription of SGLT2i or GLP-1 RA between 1 January 2018 and 30 June 2022	Electronic healthcare records out of the public health system	All-cause mortality	
			Non-fatal myocardial infarction	
			Non-fatal stroke	
			Heart failure hospitalisation	
Gorgojo-Martínez et al (2017) [31]	T2D, HbA1c >6.5%<br>Use of dapagliflozin for at least 12 months<br>eGFR >60	Use of systemic steroids	All-cause mortality	
			Severe hypoglycaemia	
			Genital infections	

			Ketoacidosis	
Horiuchi et al (2025) [26]	T2D, diagnosed at least 1 year prior to initiation of study medication<br>Age ≥18 years	T1D<br>End-stage kidney disease diagnosed within 1 year before cohort entry	Composite of all-cause mortality and hospitalisation for heart failure	
Jensen et al (2020) [32]	Danish adults<br>T2D<br>Use of metformin	-	MACE	Composite of non-fatal myocardial infarction, non-fatal stroke, and cardiovascular death
			All-cause mortality	
			Severe hypoglycaemia	
Jhu et al (2024) [33]	T2D<br>Age ≥18 years<br>eGFR ≥60 mL/min/1.73 m2,<br>Using GLP-1 RA or SGLT2i	Malignancies<br>Previous kidney transplants<br>Adult polycystic kidney disease before the index date<br>Fewer than three health-care visits<br>Changes in medication at index date	MACE	Ischaemic stroke, acute myocardial infarction, cardiac arrest, and mortality
			All-cause mortality	
			Kidney composite endpoint	end-stage kidney disease, acute kidney injury, and all-cause mortality
			Severe hypoglycaemia	
			Genito-urinary tract infections	
			Ketoacidosis	
Kobayashi et al (2023) [34]	T2D<br>≥22 years old in 2022<br>GLP1RAs for more than one year<br>Visit to Kanagawa outpatient clinics between July and October 2020	T1D<br>Requirement for chronic dialysis<br>Severe liver dysfunction<br>Terminal-stage malignancy<br>Pregnancy<br>Severe liver dysfunction<br>Intent to opt-out during the study	Kidney composite endpoint	Progression of ACR worsening or ≥15% of eGFR decline/year
Lau et al (2022) [35]	T2D<br>Age ≥18 years	-	MACE	Composite of coronary heart disease, acute myocardial infarction, other ischemic heart disease, heart failure, stroke, transient ischemic attack and peripheral vascular disease
			Heart failure hospitalisation	
			Kidney endpoint	eGFR <15 ml/min/1.73m2
			Severe hypoglycaemia	
			Ketoacidosis	
Liu et al (2025) [36]	T2D<br>Age ≥18 years<br>Hospitalised for ACS (STEMI, NSTEMI, UA)<br>Continuously treated with SGLT2-i for at least 3 months	Severe HF<br>Malignant ventricular arrhythmias<br>Perioperative cardiopulmonary resuscitation,<br>Malignant neoplasm, previous use of GLP-1 RA, prior ACS event	MACE	Composite of all-cause death, cardiovascular death, non-fatal myocardial infarction, non-fatal stroke, coronary revascularisation, and heart failure readmission
			All-cause mortality	
			Cardiovascular mortality	

			Non-fatal myocardial infarction	
			Non-fatal stroke	
			Heart failure hospitalisation	
Lopez et al (2022) [37]	T2D<br>Established cardiovascular disease (ischemic heart disease, cerebrovascular disease, or peripheral artery disease)<br>HFrEF (ejection fraction >40%)	Life expectancy <3 months attributable to malignancy or end-stage liver disease	MACE	Composite of all-cause mortality, non-fatal myocardial infarction, and non-fatal cerebrovascular event
			Nonfatal myocardial infarction	
			Nonfatal stroke	
			Heart failure hospitalisation	
Luo et al (2023) [38]	T2D	-	All-cause mortality	
			Non-fatal myocardial infarction	
			Nonfatal stroke	
Marfella et al 2024 [39]	T2D<br>first myocardial infarction and treated with GLP-1 RA or SGLT2i >3 months prior to hospitalization.	evidence of heart failure, valvular defects, malignant neoplasms, or secondary causes of hypertension	MACE	Composite of all-cause mortality, hospitalization for heart failure, and acute coronary syndrome
Patel et al (2024) [40]	Age ≥18 years<br>T2D<br>BMI≥27<br>history of HFpEF<br>LVEF ≥45%<br>using SGLT2i	-	All-cause mortality	
			Heart failure hospitalisation	
			Kidney composite endpoint	Kidney replacement therapy
			Severe hypoglycaemia	
			Gastro-intestinal side effects	
Riley et al (2023) [41]	T2D and insulin therapy	T1D	All-cause mortality	
			Nonfatal myocardial infarction	
			Nonfatal stroke	
			Kidney endpoint	Chronic kidney disease, not further specified
Schechter et al (2023) [42]	T2D<br>Start of GLP-1RA or insulin	T1D<br>eGFR <15 or kidney replacement therapy<br>Use of insulin/GLP-1 RA in the last years<br>Diagnosis of dementia, history of organ transplantation, cancer (within the past 5 years), heart failure<br>Hospitalized for ≥ 5 consecutive days within the past 180 days	Kidney endpoint	Composite of ≥ 40% eGFR reduction from baseline or new end-stage renal disease
Simms-Williams et al (2024a) [43]	Age ≥18 years<br>≥1 year of medical history in CPRD<br>GLP-1 RA user	no history of T2D at cohort entry<br>Use of SGLT2i in the past year	MACE	Composite of myocardial infarction, ischaemic stroke, and cardiovascular mortality

			All-cause mortality	
			Cardiovascular mortality	
			Nonfatal myocardial infarction	
			Nonfatal stroke	
			Kidney endpoint	Composite of acute kidney injury, chronic kidney disease, hypertensive chronic renal disease, unspecified kidney failure, and renal complications of diabetes.
Simms-Williams et al (2024b) [43]	Age ≥18 years<br>≥1 year of medical history in CPRD<br>SGLT2i user	no history of T2D at cohort entry<br>Use of GLP-1RA in the past year	MACE	Composite of myocardial infarction, ischaemic stroke, and cardiovascular mortality
			All-cause mortality	
			Cardiovascular mortality	
			Nonfatal myocardial infarction	
			Nonfatal stroke	
			Kidney endpoint	Composite of acute kidney injury, chronic kidney disease, hypertensive chronic renal disease, unspecified kidney failure, and renal complications of diabetes.
Wright et al (2022) [44]	Use of non-insulin antidiabetic medications between January 1998 and July 2018<br>Initiation of at least one new clas of antidiabetic medication (first-line inititation, switch, or addition to treatment regimen) between November 2012 and July 2018	Use of insulin<br>Women with PCOS or gestational diabetes<br>History of HIV or antiretroviral therapy<br>CVD<br>HF	MACE	Composite of myocardial infarction/acute coronary syndrome, stroke/transient ischaemic attack including intracerebral and subarachnoid haemorrhage, cardiovascular mortality
			Heart failure hospitalisation	

Abbreviatons: ACEi, ace-inhibitor; ARB, angiotensin receptor blocker; BMI, body mass index; CKD, chronic kidney disease; CPRD, Clinical Practice Research Datalink; CrCl, creatinine clearance; CVD, cardiovascular disease; CV, cardiovascular; eGFR, estimated glomerular filtration rate; GLP-1 RA, glucagon-like peptide-1 receptor agonist; HDL, high-density lipoprotein; HIV, human immunodeficiency virus; KRT, kidney replacement therapy; LDL, low-density lipoprotein; MACE, major adverse cardiovascular events; MEN-2, multiple endocrine neoplasia; NYHA, New York Heart Association; SCr, serum creatinine; SGLT2 inhibitor, sodium-glucose co-transporter-2 inhibitor; T1D, type 1 diabetes; T2D, type 2 diabetes; UACR, urinary albumin creatinine ratio.
† protocol only

ESM Table 6: Key methodology of included cohort studies

**Study (publication year)**	**Data source**	**Method to handle confounding**	**Confounders included**	**Method to handle missing data**

Chaiyakunapruk et al (2025) [29]	Komodo's Healthcare Map comprising US pharmacy en medical claims data	Entropy balancing (an alternative to IPTW based on PS)	Age, race and ethnicity, geographical region, insurance type, prescriber specialty for index drug, index year, months of SGLT2i use in the study period before the index date, months of SGLT2i use in the whole database before the index date, months since first observed T2D diagnosis before the index date, baseline SGLT2i use and adherence, CCI score excluding diabetes, MI from the CCI, peptic ulcer disease from the CCI, renal disease from the CCI, DCSI score, cardiovascular complication from the DCSI score, nephropathy from the DCSI CKD stage, number of outpatient visits at baseline, number of outpatient visits 60 days before the index date, number of T2D-related outpatient visits 60 days before the index date, number of T2D-related inpatient visits 60 days before the index date, comorbidities, depression, acute pancreatitis, chronic pancreatitis, obesity, gastroesophageal reflux disease, medications, number of glucose-lowering therapies used, metformin use, SU use, DPP4i use, basal insulin use, other insulin use, other diuretic use, procedures, PCI, HbA1c and BMI measures, baseline HbA1c, baseline BMI, baseline Hb A1c category, baseline BMI category, T2D with ASCVD cohort, months since first observed ASCVD diagnosis before the index date, type 2 diabetes with CKD cohort, months since first observed CKD diagnosis before the index date.	Not reported. Patients with missing data on age and sex were excluded
Dave et al (2021) [30]	US insurance claims from three databases (Optium Clinformatics Data Mart Database; IBM Marketscan; Medicare components of Optum)	Propensity score matching	Demographics and calendar time (e.g. age, sex and calendar year of cohort entry), complications of diabetes (e.g. diabetic-neuropathy, nephropathy, retinopathy), oral and injectable antidiabetic therapy (e.g. metformin, insulin, DPP-4i), cardiovascular conditions (e.g. myocardial infarction, stroke, HF), cardiovascular medications (e.g. dispensing of BB, loop diuretics, statins), non-cardiovascular comorbid conditions (e.g. diagnosis of CKD, COPD, psychiatric conditions), non-cardiovascular medications (e.g. dispensing of anticonvulsants, antidepressants), and measures of burden of comorbidities and healthcare utilization (e.g. combined comorbid index, number of hospitalizations, number of medications)	Apart from HbA1c there were no missing data. HbA1c was not included in propensity score
García-Vega et al (2024) [25]	Integrated electronic medical records from primary care and hospitals obtained through "big data" technologies in a healthy area in Galicia	Adjusted Cox models	Demographic (age, sex) and known risk factors (dyslipidaemia, CAD, AF, HF, cerebrovascular accident), therapy duration, and cardiovascular risk factors (high blood pressure, obesity or overweight, and dyslipidaemia)	Not reported
Gorgojo-Martínez et al (2017) [31]	Retrospective cohort of real-world data Hospital Universitario Fundación Alcorcón	Multivariable regression analysis	Age, gender, duration of T2D, baseline A1C, BMI, and eGFR	Not reported
Horiuchi et al 2025 [26]	TriNetX Global Research Network (EHR data from US health care institutions)	Propensity score matching	D64 Other anaemias, E11.0 Type 2 DM with hyperosmolarity, E11.1 T2D with ketoacidosis, E11.2 T2D with kidney complications, E11.3 T2D with ophthalmic complications, E11.4 T2D with neurological complications, E11.5 T2D with circulatory complications, E11.64 T2D with hypoglycaemia, E11.65 T2D with hyperglycaemia, E87.5 Hyperkalaemia, E87.6 Hypokalaemia, F31 Bipolar disorder, F32 Depressive episode, F33 Major depressive disorder, recurrent, G30 Alzheimer's disease, G31 Other degenerative diseases of the nervous system, not elsewhere classified, G47.3 Sleep apnoea, I10 Hypertension, I11 Hypertensive heart disease, I12 Hypertensive CKD, I13 Hypertensive heart and CKD, I20 Angina pectoris, I21 Acute MI, I25 Chronic IHD, I27.2 Other secondary pulmonary hypertension, I34 Nonrheumatic mitral valve disorders, I35 Nonrheumatic aortic valve disorders, I36 Nonrheumatic tricuspid valve disorders, I37 Nonrheumatic pulmonary valve disorders, I42 Cardiomyopathy, I48 Atrial fibrillation and flutter, I49 Other cardiac arrhythmias, I50 HF, I50.2 Systolic (congestive) HF, I50.3 Diastolic (congestive) HF, I50.4 Combined systolic (congestive) and diastolic (congestive) HF, I63 Cerebral infarction, I65 Occlusion and stenosis of precerebral arteries, not resulting in cerebral infarction, I70 Atherosclerosis, I73.9 PAD, unspecified, I82 Other venous embolism and thrombosis, I95 Hypotension, J18 Pneumonia, unspecified organism, J44.COPD, unspecified, M62.84 Sarcopenia, N17 AKI, N18 CKD, N18.1 CKD, stage 1, N18.2 CKD, stage 2, N18.3 CKD, stage 3, N18.4 CKD, stage 4, N18.5 CKD, stage 5, R54 Age-related physical debility, R60 Oedema, not elsewhere classified, R64 Cachexia, Z72.0 Tobacco use, Age; years, Male; n (%), Race; n (%), White, Black, Asian, American Indian or Alaska Native, Native Hawaiian or Other Pacific Islander, Hispanic or Latino, Other Race, Hypertension (I10),	Not reported

			Hypertensive heart disease (I11), Hypertensive CKD (I12), Hypertensive heart and CKD (I13), Acute MI (I21), Angina pectoris (I20), Chronic IHD (I25), Cardiomyopathy (I42), Nonrheumatic aortic valve disorders (I35), Nonrheumatic mitral valve disorders (I34), Nonrheumatic tricuspid valve disorders (I36), Nonrheumatic pulmonary valve disorders (I37), Atrial fibrillation and flutter (I48), Other cardiac arrhythmias (I49), Cerebral infarction (I63), Occlusion and stenosis of precerebral arteries (I65), PAD (I73.9), Atherosclerosis (I70), Sleep apnoea (G47.3), Tobacco use (Z72.0), AKI (N17), CKD (N18), CKD stage 1 (N18.1), CKD stage 2 (N18.2), CKD stage 3 (N18.3), CKD stage 4 (N18.4), CKD stage 5 (N18.5), Anaemia (D64), COPD (J44.9), Pneumonia (J18), Secondary pulmonary hypertension (I27.2), Hypokalaemia (E87.6), Hyperkalaemia (E87.5), Hypotension (I95), Oedema (R60), Venous embolism and thrombosis (I82), Age-related physical debility (R54), Cachexia (R64), Sarcopenia (M62.84), Alzheimer's disease (G30), Bipolar disorder (F31), Depressive episode (F32), Major depressive disorder recurrent (F33), Other degenerative diseases of the nervous system (G31), T2D with neurological complications (E11.4), T2D with kidney complications (E11.2), T2D with ophthalmic complications (E11.3), T2D with circulatory complications (E11.5), T2D with hyperosmolarity (E11.0), T2D with ketoacidosis (E11.1), T2D with hyperglycaemia (E11.65), T2D with hypoglycaemia (E11.64), SBP, Heart Rate, BMI; HbA1c; eGFR; Sodium, Potassium, Haematocrit, Haemoglobin, BNP, NT-proBNP, LVEF, Insulins, Biguanides, SUs, SGLT2i, TZD, Alpha glucosidase inhibitors, BBs, ACEi, ARB, Sacubitril, MRAs, Loop diuretics, Thiazides, Digoxin, Organic nitrates, CCBs, Hydralazine, Platelet aggregation inhibitors, VKAs, DOACs, Heparin, statins Fibrates, Other lipid modifying agents, NSAIDs, Opioid analgesics, Non-opioid analgesics	
Jensen et al (2020) [32]	Danish National Patient Registry (including primary and secondary care)	Adjusted Cox models	Age, sex, diabetes duration, history of CKD, history of nonfatal MACE prior to baseline excluding cardiovascular deaths, treatment start date, highest completed education, and income.	Not reported
Jhu et al (2024) [33]	TriNetX Global Research Network (EHR data from US health care institutions)	Propensity score matching	Hypertensive diseases, disorders of lipoprotein metabolism and other lipidaemias, unspecified PAD, HF, IHD, cerebrovascular diseases, liver fibrosis and cirrhosis, other COPD, other anxiety disorders, and gout; is treated with insulin, oral hypoglycaemic agents, BBs or related drugs, antilipemic agents, antiarrhythmics, ACEi, ARB, CCBs, and diuretics; and has laboratory results encompassing BMI, LDL cholesterol, triglycerides, serum/plasma/blood creatinine, blood haemoglobin, eGFR, UACR, and HbA1c.	Not reported
Kobayashi et al (2023) [34]	Electronic healthcare records from 22 primary care medical facilities in Japan (Kanawaga prefecture)	Propensity score matching	Age, sex, body weight, HbA1C, SBP, DBP, eGFR, ln(UACR), concomitant use of other glucose-lowering agents and statins	Patients with missing ACR were excluded. No other methods reported
Lau et al (2022) [35]	IMRD, a database comprising anonymized electronic primary health care records for 15 million patients from >750 general practices across the United Kingdom.	IPTW	Age, sex, smoking status, drinking status, duration of T2D, duration of SGLT2i prescription, anthropometric and clinical measurements, laboratory readings, drug prescription within 1 year, and comorbidity status at baseline. Baseline BMI, fasting glucose, HbA1c, average SBP and DBP within 1 year before baseline, total cholesterol to HDL-cholesterol ratio, LDL-cholesterol, and triglycerides	Multiple imputation
Liu et al (2025) [36]	Electronic healthcare records from a tertiary care hospital in China	Propensity score matching	Age, sex, smoking status, BMI, duration of diabetes, hypertension, chronic HF, hyperlipidaemia, HbA1c, hs-CRP, LDL, BNP, eGFR, peak cTnI, UACR, insulin, ACEI/ARB, BBs, statins, aspirin, P2Y12i, ACS type, extent of CAD, LVEF	Not reported
Lopez et al (2022) [37]	The National veterans affairs database	Propensity score matching	Age, gender (exact), ejection fraction, glycated HbA1c, systolic blood pressure, presence of CAD, presence of PAD, Elixhauser mortality score, and Elixhauser readmission score	Patients with missing data were excluded
Luo et al (2023) [38]	prospectively collected electronic health records using the Clinical Data Analysis and Reporting System (CDARS) by the Hospital Authority (HA) of	Propensity score matching	Demographics, prior anti-diabetic drugs, number of prior anti-diabetic drugs, prior comorbidities, renal function, duration from T2DM diagnosis initial drug exposure, HbA1c and fasting glucose	Not reported

	Hong Kong (public hospitals, their outpatient clinics, and ambulatory and day-care facilities in Hong Kong)			
Marfella et al (2024) [39]	Prospective data from various Italian hospitals	Multivariable analysis	Age, sex, BMI, diabetes duration, glycaemic control, LDL-cholesterol, triglycerides, troponin, creatinine, MLD, the prevalence of STEMI, hypertension, dyslipidaemia, and smoking	Not reported
Patel et al (2024) [40]	TriNetX Global Research Network (EHR data from US health care institutions)	Propensity score matching	Variables selected for assessment were based on their potential impact on overall and cardiovascular outcomes.	Not reported
Riley et al (2023) [41]	TriNetX Global Research Network (EHR data from US health care institutions)	Propensity score matching	Age, gender, presence of IHD, hypertension, HF, CKD, HbA1c	Not reported
Schechter et al (2023) [42]	Maccabi Healthcare Services	Propensity score matching	Demographics: Age, sex, socioeconomic status (1-3, 4-5, 6-7, 8-10), time of entering into study (by year and quartiles), body mass index (<25, 25-<30, 30-<35, 35-<40, ≥40, missing; kg/m2), smoking status (current smoker, past smoker, never smoker, missing), SBP, DBP. Co-morbidities: duration (years) with diabetes (≤2, 2-5, 5-10, ≥10), IHD, MI or after cardiac revascularization procedure, unstable angina, stable angina, AF, cerebrovascular disease, stroke, TIA, PAD, bariatric surgery, hypertension, hyperlipidaemia, cancer, diabetic neuropathy, diabetic nephropathy, diabetic retinopathy or other ophthalmic manifestations, diabetic foot or lower extremity amputation, liver disease, osteoarthritis, obstructive sleep apnoea, COPD, hypothyroidism, anxiety, depression, psychoses. Medications: metformin, SUs, TZD, meglitinides, alpha-glucosidase inhibitors, SGLT2i, fast-acting insulin, ACEi, ARB, antihypertensive drugs, CCBs, thiazides, loop diuretics, other diuretics, nitrates, other hypertension drugs, statins, PCSK-9 inhibitors, other lipid-lowering drugs (excluding statins), COPD or asthma medications, antiplatelet, anticoagulants, heparin and low-molecular weight heparins, oral corticosteroids, NSAIDs, opioids, antidepressants, antipsychotics, anti-Parkinson's agents, MRAs, digoxin, anti-arrhythmic drugs, bisphosphonates, anti-convulsants, benzodiazepines, PPIs. Laboratory examinations: eGFR, UACR; below detectable, <15, 15-<30, 30-300, ≥300, missing), total cholesterol, LDL cholesterol, HDL cholesterol, triglycerides, baseline eGFR slope, Fasting-plasma glucose, Alanine transaminase (ALT), aspartate transaminase (AST), alkaline phosphatase, blood urea nitrogen, platelets count, haemoglobin, serum albumin, sodium, potassium, calcium	A missing values category was included in the variables for the propensity score
Simms-Williams et al (2024) [43]	UK Clinical Practice Research Datalink linked to Hospital Episode Statistics Admitted Patient Care and Office for National Statistics databases	Propensity score matching	Age (modelled as a continuous variable using cubic splines with five knots at the 5th, 27.5th, 50th, 72.5th, and 95th centiles), sex, smoking status, BMI, alcohol related disorders, and cohort entry year (2013-15, 2016-18, 2019-21). We also considered proxies for severity of diabetes, including duration of diabetes (calculated by the time difference between cohort entry date and date of the first of either a HbA1c >6.4%, a diagnosis of T2D, or prescription for an antihyperglycaemic drug ever before cohort entry), HbA1c level (≤7.0%, 7.1-8.0%, or >8.0%), types of antihyperglycaemic drugs used in the year before cohort entry (metformin, SUs, TZDs, meglitinides, α-glucosidase inhibitors, DPP4i, and insulin), microvascular (nephropathy, neuropathy, retinopathy) and macrovascular complications of diabetes (MI, ischaemic stroke, PAD, CAD, PCI, HF, all measured in ever before study cohort entry). Additionally, we considered common comorbidities (cancer (other than non-melanoma skin cancer), AF, thyroid diseases, and COPD), as well as common prescription drugs (antihypertensives (diuretics, BBs, CCBs, ACEis, ARBs, and others), NSAIDs, paracetamol, acetylsalicylic acid, other antiplatelet agents, statins, fibrates, digoxin, opioids), and markers, of healthcare seeking behaviour (colorectal cancer screening, prostate specific antigen screening, and influenza vaccination)	An unknown category was included for variables containing missing data, which was included in the propensity score
Wright et al (2022) [44]	Clinical Practice Research Datalink (CPRD) GOLD, GP practices, Clinical Practice Research Datalink (CPRD)	Matching age (±2 years), date of cohort entry (±1 year), and duration of treated diabetes	Matching: (age, duration of treated diabetes), clinical characteristics (ethnicity, IMD, microvascular complications, CCI, smoking status, BMI, HbA1c, blood pressure, total cholesterol), and drug history (prescriptions for ADMs, antihypertensive agents, lipid-lowering agents, antiplatelet agents, corticosteroids, NSAIDs, and anticoagulants in the year before cohort entry; ever exposure to ADMs; number of ADMs prescribed before cohort entry)sex, age, date of cohort entry, duration of diabetes	Missing category was used for propensity scores. No other method for missing data was reported

	Aurum, GP practices, SAIL, GP practices	(±1 year). A sensitivity analyses was performed using propensity scores	PMC: age, gender, region, IMD, ethnicity, duration of T2D, duration of treated diabetes, history of smoking, current smoker at cohort entry, history of renal disease, microvascular disease (nephropathy, retinopathy, neuropathy), cardiovascular disease, AF, COPD, dementia, liver disease, cancer, depression, bipolar disorder, schizophraenia, mean value over patient history and closest value in the year prior to cohort entry of HbA1c, BMI, SBP, DBP, total cholesterol, creatinine, eGFR and binary variables indicating prescription for the following drugs in the year prior to cohort entry: antidiabetic medications (metformin, SU, TZD, acarbose, meglitinides, GLP-1RA, DPP-4i, SGLT2i), antihypertensive agents (ACI, ARB, BB, CCB, diuretics, other), lipid lowering agents (statins, fibrates, ezetimibe, other), antiplatelet agents (aspirin, clopidogrel, others), NSAIDs, steroids, anticoagulants, antipsychotics, anti-osteoporotic agents	

ACEi, Angiotensin-Converting Enzyme inhibitor; ACS, Acute Coronary Syndrome; ADM, Antidiabetic Medication; AF, Atrial Fibrillation; AKI, Acute Kidney Injury; ALT, Alanine Transaminase; ARB, Angiotensin II Receptor Blocker; ASCVD, atherosclerotic cardiovascular disease; AST, Aspartate Transaminase; BB, Beta Blocker; BMI, Body Mass Index; BNP, B-type Natriuretic Peptide; CAD, Coronary Artery Disease; CCB, Calcium Channel Blocker; CCI, Charlson Comorbidity Index; CDARS, Clinical Data Analysis and Reporting System; CKD, Chronic Kidney Disease; COPD, Chronic Obstructive Pulmonary Disease; CPRD, Clinical Practice Research Datalink; cTnI, Cardiac Troponin I; DBP, Diastolic Blood Pressure; DCSI, Diabetes Complications Severity Index; DOAC, direct oral anticoagulant; DPP4i, Dipeptidyl Peptidase-4 Inhibitor; eGFR, Estimated Glomerular Filtration Rate; HER, Electronic Health Record; GLP-1RA, Glucagon-Like Peptide-1 Receptor Agonist; HbA1c, Haemoglobin A1c; HA, Hospital Authority (Hong Kong); HF, Heart Failure; HDL, high-density lipoprotein; hs-CRP, High-Sensitivity C-Reactive Protein; IHD, ischaemic heart disease; IMD, Index of Multiple Deprivation; IMRD, IQVIA Medical Research Data; IPTW, Inverse Probability of Treatment Weighting; LDL, Low-Density Lipoprotein; LVEF, Left Ventricular Ejection Fraction; MACE, Major Adverse Cardiovascular Events; MI, Myocardial Infarction; MLD, Mean Lesion Diameter; MRA, Mineralocorticoid Receptor Antagonist; NSAID, Non-Steroidal Anti-Inflammatory Drug; NT-proBNP, N-terminal pro B-type Natriuretic Peptide; PAD, Peripheral Artery Disease; PCI, Percutaneous Coronary Intervention; PCSK-9, Proprotein Convertase Subtilisin/Kexin Type 9; PMC, Propensity Matched Cohort; PS, Propensity Score; PSM, Propensity Score Matching; P2Y12i, P2Y12 Inhibitor; SAIL, Secure Anonymised Information Linkage; SBP, Systolic Blood Pressure; SGLT2i, Sodium-Glucose Cotransporter 2 Inhibitor; SU, Sulfonylurea; T2D, Type 2 Diabetes; TIA, Transient Ischaemic Attack; TZD, Thiazolidinedione; UACR, Urine Albumin-to-Creatinine Ratio; US, United States; VKA, vitamin K antagonist

ESM Table 7: Risk of bias of included cohort studies

Study	Confounding*	Selection of participants	Classification of interventions	Deviations from the intended interventions	Missing data	Measurement of outcome	Selective outcome reporting	Overall risk of bias
Chaiyakunapruk et al (2025) [29]	Low	Low	Low	No information – no information on time on treatment and intercurrent events	Serious – Participants excluded due to missing data on age and sex	Low	Low	Serious risk of bias due to risk of bias in missing data
Dave et al (2021) [30]	Serious – results not adjusted for albuminuria	Low	Low	No information – no information on time on treatment and intercurrent events	Low	Low	Low	Serious risk of bias due risk of bias in confounding
García-Vega et al (2024) [25]	Serious – results not adjusted for smoking, hypertension, HbA1c or diabetes duration, SCr or eGFR. BMI, and albuminuria	No information – due to vague description of intervention unclear whether prevalent user bias occurred in combination group	Serious – combination therapy arm not clearly described	No information – no information on time on treatment and intercurrent events	Serious – no method of handling missing data specified in methods. Assumption primary analysis is a complete case analysis	Low	Low	Serious risk of bias due to risk of bias in confounding, classification of intervention status, and missing data
Gorgojo-Martínez et al (2017) [31]	Serious – results not adjusted for smoking, hypertension, prior CVD, hyperlipidaemia, and albuminuria	Low	Low	Serious – significant baseline differences use of in insulin and other antidiabetic and cardiovascular medications and cardiovascular, not adjusted for in analysis	Serious – no method of handling missing data specified in methods. Assumption primary analysis is a complete case analysis	Low	Low	Serious risk of bias due to risk of bias in confounding, deviations from intended interventions, and missing data
Horiuchi et al 2025 [26]	Serious – results not adjusted for hyperlipidaemia or lipids, BMI, or albuminuria	Low	Low	No information – no information on time on treatment and intercurrent events	Serious – no method of handling missing data specified in methods. Assumption primary analysis is a complete case analysis	Low	Low	Serious risk of bias due to risk of bias in confounding and missing data
Jensen et al (2020) [32]	Serious – results not adjusted for blood pressure/hypertension, BMI, comedication, cholesterol, eGFR, albuminuria, or smoking)	Low	Low	No information – no information on time on treatment and intercurrent events	Serious – no method of handling missing data specified in methods. Assumption primary analysis is a complete case analysis	Low	Low	Serious risk of bias due to issues with confounding and missing data
Jhu et al (2024) [33]	Low	Low	Low	No information – no information on time on treatment and intercurrent events	Serious – no method of handling missing data specified in methods. Assumption primary analysis is a complete case analysis	Low	Low	Serious risk of bias due to issues with handling of missing data
Kobayashi et al (2023) [34]	Serious – results not adjusted for smoking, BMI/waist circumference, history of CVD	Critical – probably prevalent user bias	Low	No information – no information on time on treatment and intercurrent events	Serious – Patients excluded due to missing data on covariates.	Low	Low	Critical risk of bias due to issues with critical issues due to selection of participants and issues due to confounding and missing data
Lau et al (2022) [35]	Low	Low	Low	No information – no information on time on treatment and intercurrent events	Low	Low	Low	Low risk of bias
Liu et al (2025) [36]	Low	Moderate – Unclear if start of FU and start of GLP-1 RA coincide	Low	No information – no information on time on	Serious – no method of handling missing data specified in methods.	Low	Low	Serious risk of bias due to moderate issues with selection of participants

				treatment and intercurrent events	Assumption primary analysis is a complete case analysis			and handling of missing data
Lopez et al (2022) [37]	Serious – results not adjusted for smoking, BMI, CKD, SCr, or eGFR, and albuminuria	Low	Low	Serious – significant differences in baseline insulin use, not adjusted for in analysis	Serious – participants were excluded based on missing covariates	Low	Low	Serious risk of bias due to risk of bias in confounding, deviations from intended interventions, and missing data
Luo et al (2023) [38]	Serious – results not adjusted for smoking and albuminuria and unclear which if hypertension and hyperlipidaemia were included in comorbidities score. Overadjustment for confounding with propensity scores and multivariable adjustment	No information – not specified if participants were prevalent or incident users	Serious – description of intervention and control group lacking completely	No information – description of intervention and control group completely lacking	Serious – no method of handling missing data specified in methods. Assumption primary analysis is a complete case analysis	Low	Serious – outcomes specified in methods not reported in results. All results reported in manuscript not specified in method	Serious risk of bias due to risk of bias in confounding, classification of interventions, missing data, and selective outcome reporting
Marfella et al (2024) [39]	Serious – adjustment for HbA1c during follow-up and no adjustment for albuminuria	Critical – participants are assigned to intervention and control group based on baseline HbA1c. Control arm comprises prevalent users	Low	No information – no information on time on treatment and intercurrent events	Serious – no method of handling missing data specified in methods. Assumption primary analysis is a complete case analysis	Low	Low	Critical risk of bias due to confounding, selection of participants, deviations from intended interventions, and missing data
Patel et al (2024) [40]	Serious – unclear for which confounders results were adjusted for. At least smoking and albuminuria are missing based on the baseline table	Low	Low	No information – no information on time on treatment and intercurrent events	Serious – no method of handling missing data specified in methods. Assumption primary analysis is a complete case analysis	Low	Serious – all-cause mortality not reported in methods as outcome	Serious risk of bias due to issues with confounding, missing data, and selection of reported results
Riley et al (2023) [41]	Serious – no adjustment for hyperlipidaemia, albuminuria, and BMI	Low	Low	No information – no information on time on treatment and intercurrent events	Serious – no method of handling missing data specified in methods. Assumption primary analysis is a complete case analysis	Low	Low	Serious risk of bias due to confounding and missing data
Schechter et al (2023) [42]	Low	Low	Low	No information – no information on time on treatment and intercurrent events	Moderate - propensity matching with category for missingness may not have removed bias due to missing data	Low	Low	Moderate risk of bias due to missing data
Simms-Williams et al (2024) [43]	Serious – no adjustment for albuminuria	Low	Low	No information – no information on time on treatment and intercurrent events	Moderate – propensity matching with category for missingness may not have removed bias due to missing data	Low	Low	Serious risk of bias due to confounding
Wright et al (2022) [44]	Serious – results not adjusted for hypercholesterolaemia and albuminuria	Low	Low	Serious – important differences baseline differences in medication use a microvascular complications, although analysis is adjusted for most variables	Serious – no method of handling missing data specified in methods. Assumption primary analysis is a complete case analysis	Low	Low	Serious risk of bias due to confounding, deviations from the intended interventions, and missing data

* to be rated as low risk of bias, studies had to adequately adjust for sex, age or duration of diabetes, HbA1c, hypertension or blood pressure, hyperlipidaemia or lipids, history of cardiovascular disease (CVD), serum creatinine (SCr), estimated glomerular filtration rate (eGFR) or history of chronic kidney disease (CKD), urinary albumin creatin ratio (UACR) or albuminuria, smoking, and body mass index (BMI) or waist circumference.

ESM Table 8: Risk of severe hypoglycaemia of combining a SGLT2 inhibitor and a GLP-1 RA compared to SGLT2 inhibitor or GLP-1 RA monotherapy

	Combination therapy		Monotherapy	
**Study**	**Events (%)**	**Total**	**Events (%)**	**Total**
**Observational studies**				
Gorgojo-Martínez et al (2017) [31]	2 (2%)	109	1 (1%)	104
Jhu et al (2024) [33]	2912 (5%)	62,214	2098 (3%)	64,100
Lau et al (2022) [35]	15 (1%)	1461	23 (2%)	1427
Patel et al (2024) [40]	146 (2%)	7044	159 (2%)	7044
**Total**	3075 (4%)	70,828	2284 (3%)	72,657

ESM Table 9: Risk of diabetic ketoacidosis of combining a SGLT2 inhibitor and a GLP-1 RA compared to SGLT2 inhibitor or GLP-1 RA monotherapy

	Combination therapy		Monotherapy	
**Study**	**Events (%)**	**Total**	**Events (%)**	**Total**
Gorgojo-Martínez et al (2017) [31]	0 (0%)	109	0 (0)	104
Jhu et al (2024) [33]	261 (<1%)	68,216	259 (<1%)	68,161
Lau et al. (2022) [35]	2 (<1%)	1461	2 (<1%)	1427
**Total**	263 (<1%)	69,786	261 (<1%)	69,692

ESM Table 10: Risk of genito-urinary tract infections of combining a SGLT2 inhibitor and a GLP-1 RA compared to SGLT2 inhibitor or GLP-1 RA monotherapy

	Combination therapy		Monotherapy	
**Study**	**Events (%)**	**Total**	**Events (%)**	**Total**
Gorgojo-Martínez et al (2017) [31]	3 (3%)	109	2 (2%)	104
Jhu et al (2024) [33]	2410	61,089	2295	61,671
**Subtotal**	2413 (4%)	61,198	2297 (4%)	61,775

ESM Table 11: Risk of gastro-intestinal side effects of combining a SGLT2 inhibitor and GLP-1 RA compared to SGLT2 inhibitor or GLP-1 RA monotherapy

	Combination therapy		Monotherapy	
**Study**	**Events (%)**	**Total**	**Events (%)**	**Total**
Patel et al (2024) [40]	2128 (30%)	7044	2159 (31%)	7044
**Subtotal**	2128 (30%)	7044	2159 (31%)	7044

ESM Figure 1: flow chart of search

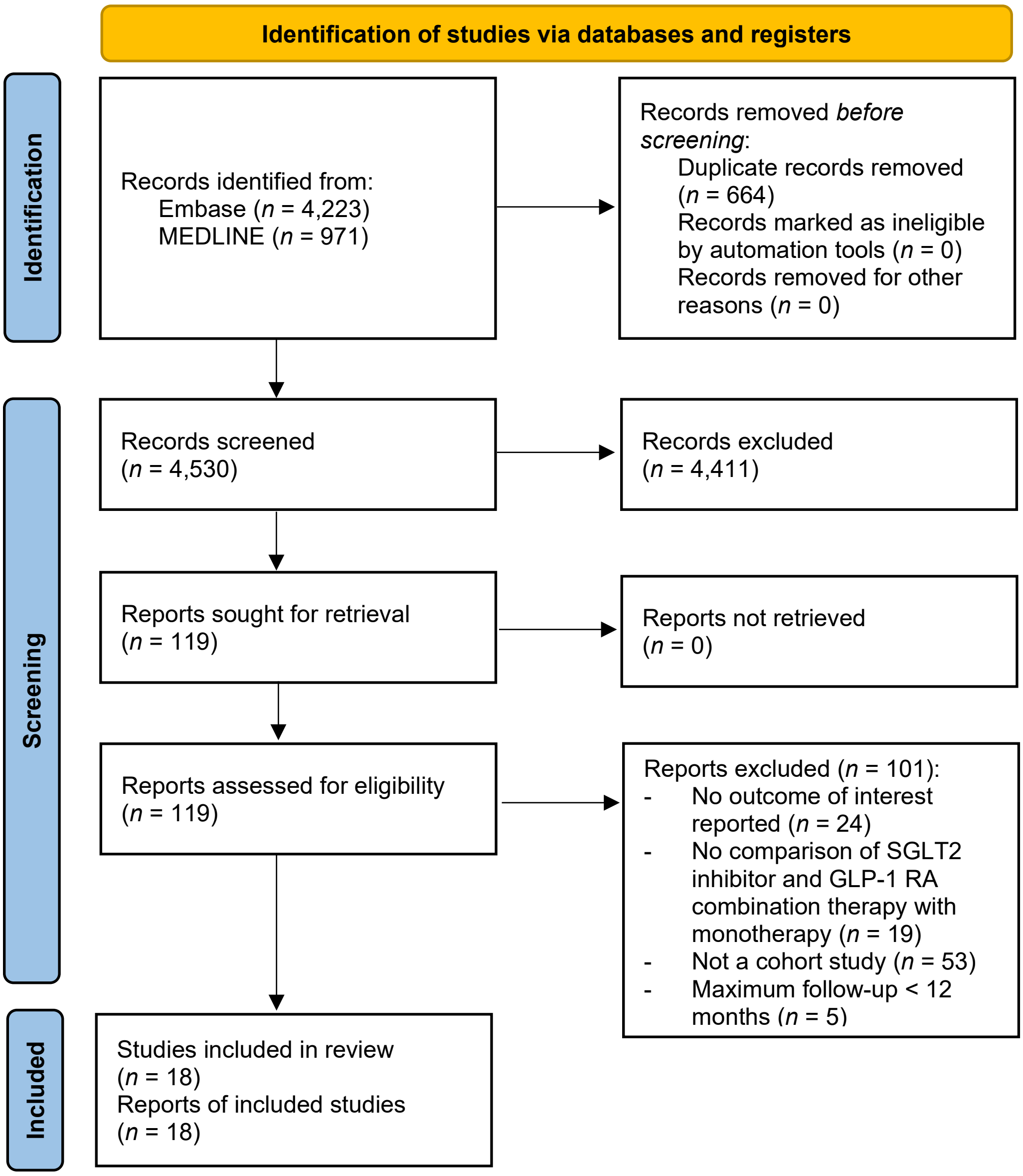

ESM Figure 2: Risk of bias summary of included cohort studies

Risk of bias domains

Study	D1	D2	D3	D4	D5	D6	D7	Overall
Chaiyakunapruk et al 2025	+	+	+	?	X	+	+	X
Dave et al 2021	X	+	+	?	+	+	+	X
García-Vega et al 2024	X	+	X	?	X	+	+	X
Gorgojo-Martínez et al 2017	X	+	+	X	X	+	+	X
Horiuchi et al 2025	X	+	+	?	X	+	+	X
Jensen et al 2020	X	+	+	?	X	+	+	X
Jhu et al 2024	+	+	+	?	X	+	+	X
Kobayashi et al 2023	X	!	+	?	X	+	+	!
Lau et al 2022	+	+	+	?	+	+	+	+
Liu et al 2025	+	-	+	?	X	+	+	X
Lopez et al 2022	X	+	+	X	X	+	+	X
Luo et al 2023	X	?	X	?	X	+	X	X
Marfella et al 2024	X	!	+	?	X	+	+	!
Patel 2024	X	+	+	?	X	+	X	X
Riley et al 2023	X	+	+	?	X	+	+	X
Schechter et al 2023	+	+	+	?	-	+	+	-
Simms-Williams et al 2024	X	+	+	?	-	+	+	X
Wright et al 2022	X	+	+	X	X	+	+	X

Domains:
D1: Bias due to confounding.
D2: Bias due to selection of participants.
D3: Bias in classification of interventions.
D4: Bias due to deviations from intended interventions.
D5: Bias due to missing data.
D6: Bias in measurement of outcomes.
D7: Bias in selection of the reported result.

Judgement
! Critical
X Serious
- Moderate
+ Low
? No information

ESM Figure 3: Effect of combining a SGLT2 inhibitor and a GLP-1 RA compared to SGLT2 inhibitor or GLP-1 RA monotherapy on major adverse cardiovascular events stratified by diabetes duration

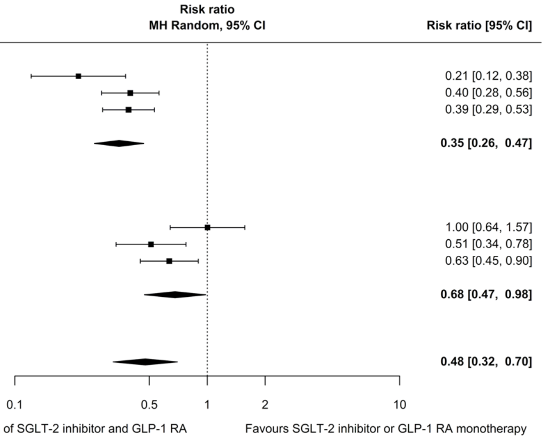

Study	Combination therapy Events	Combination therapy Total	Monotherapy Events	Monotherapy Total	Weight	Risk ratio [95% CI]
**Baseline diabetes duration >10 years**						
Marfella et al (2024) [39]	13	214	65	229	14.2%	0.21 [0.12, 0.38]
Simms-Williams et al (2024a) [43]	45	6696	113	6696	17.6%	0.40 [0.28, 0.56]
Simms-Williams et al (2024b) [43]	55	8942	141	8942	18.1%	0.39 [0.29, 0.53]
**Subtotal (95% CI)**	113	**15852**	319	**15867**	**49.9%**	**0.35 [0.26, 0.47]**
Heterogeneity: $Tau^2 = 0.03$; $Chi^2 = 3.84$, df = 2 (p= 0.15); $I^2 = 43\%$						
Test for overall effect: Z = -7.01 (p< 0.01)						
**Baseline diabetes duration ≤10 years**						
Lau et al (2022) [35]	38	1461	37	1427	16.1%	1.00 [0.64, 1.57]
Jensen et al (2020) [32]	23	1823	367	14841	16.5%	0.51 [0.34, 0.78]
Liu et al (2025) [36]	40	208	63	208	17.6%	0.63 [0.45, 0.90]
**Subtotal (95% CI)**	101	**3492**	467	**16476**	**50.2%**	**0.68 [0.47, 0.98]**
Heterogeneity: $Tau^2 = 0.06$; $Chi^2 = 4.86$, df = 2 (p= 0.09); $I^2 = 60\%$						
Test for overall effect: Z = -2.06 (p= 0.04)						
**Total (95% CI)**	214	**19344**	786	**32343**	**100%**	**0.48 [0.32, 0.70]**
Heterogeneity: $Tau^2 = 0.19$; $Chi^2 = 23.73$, df = 5 (p< 0.01); $I^2 = 83\%$						
Test for overall effect: Z = -3.77 (p< 0.01)						
Test for subgroup differences: $Chi^2 = 7.67$, df = 1 (p= 0.01)						

ESM Figure 4: Effect of combining a SGLT2 inhibitor and a GLP-1 RA compared to SGLT2 inhibitor or GLP-1 RA monotherapy on major adverse cardiovascular events stratified by baseline HbA1c

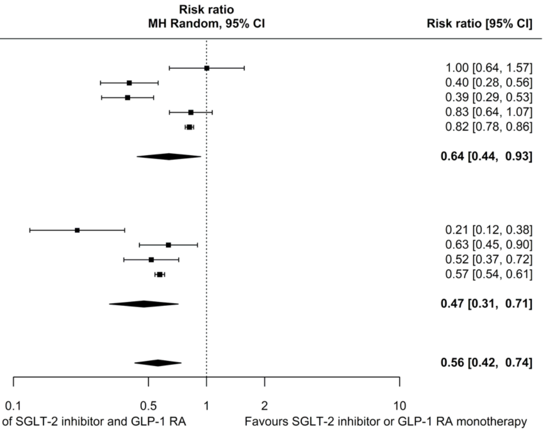

Study	Combination therapy Events	Combination therapy Total	Monotherapy Events	Monotherapy Total	Weight	Risk ratio [95% CI]
**Baseline HbA1c >64 mmol/mol**						
Lau et al (2022) [35]	38	1461	37	1427	9.7%	1.00 [0.64, 1.57]
Simms-Williams et al (2024a) [43]	45	6696	113	6696	10.9%	0.40 [0.28, 0.56]
Simms-Williams et al (2024b) [43]	55	8942	141	8942	11.2%	0.39 [0.29, 0.53]
Dave et al (2021) [30]	107	12584	129	12584	11.8%	0.83 [0.64, 1.07]
Jhu et al (2024) [33]	2635	61438	3181	60480	13.0%	0.82 [0.78, 0.86]
**Subtotal (95% CI)**	2880	**91121**	3601	**90129**	**56.6%**	**0.64 [0.44, 0.93]**
Heterogeneity: $Tau^2 = 0.16$; $Chi^2 = 37.87$, df = 4 (p< 0.01); $I^2 = 92\%$						
Test for overall effect: Z = -2.31 (p= 0.02)						
**Baseline HbA1c ≤64 mmol/mol**						
Marfella et al (2024) [39]	13	214	65	229	8.5%	0.21 [0.12, 0.38]
Liu et al (2025) [36]	40	208	63	208	10.8%	0.63 [0.45, 0.90]
Lopez et al (2022) [37]	45	343	87	343	11.1%	0.52 [0.37, 0.72]
Chaiyakunapruk et al (2025) [29]	1291	34690	8468	130220	13.0%	0.57 [0.54, 0.61]
**Subtotal (95% CI)**	1389	**35455**	8683	**131000**	**43.4%**	**0.47 [0.31, 0.71]**
Heterogeneity: $Tau^2 = 0.14$; $Chi^2 = 12.21$, df = 3 (p< 0.01); $I^2 = 88\%$						
Test for overall effect: Z = -3.57 (p< 0.01)						
**Total (95% CI)**	4269	**126576**	12284	**221129**	**100%**	**0.56 [0.42, 0.74]**
Heterogeneity: $Tau^2 = 0.15$; $Chi^2 = 130.14$, df = 8 (p< 0.01); $I^2 = 97\%$						
Test for overall effect: Z = -4.10 (p< 0.01)						
Test for subgroup differences: $Chi^2 = 1.09$, df = 1 (p= 0.30)						

ESM Figure 5: Effect of combining a SGLT2 inhibitor and a GLP-1 RA compared to SGLT2 inhibitor or GLP-1 RA monotherapy on major adverse cardiovascular events stratified by baseline history of CVD, CKD or heart failure (very high risk)

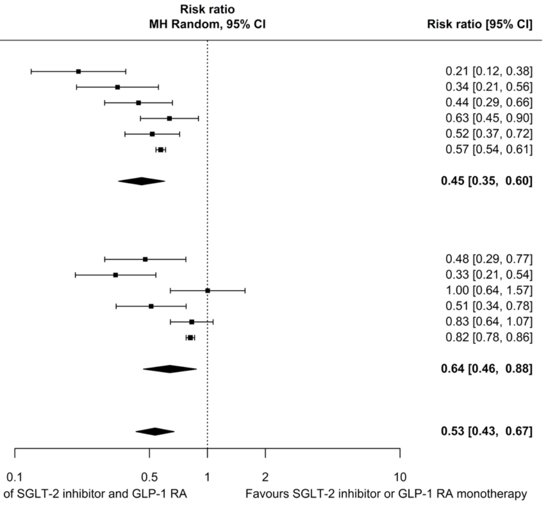

Study	Combination therapy Events	Combination therapy Total	Monotherapy Events	Monotherapy Total	Weight	Risk ratio [95% CI]
**History of CVD, CKD, or heart failure**						
Jhu et al (2024) [33]	13	214	65	229	6.5%	0.21 [0.12, 0.38]
Marfella et al (2024) [39]	21	1821	63	1865	7.2%	0.34 [0.21, 0.56]
Simms-Williams et al (2024a) [43]	33	2416	75	2410	8.0%	0.44 [0.29, 0.66]
Simms-Williams et al (2024b) [43]	40	208	63	208	8.6%	0.63 [0.45, 0.90]
Lopez et al (2022) [37]	45	343	87	343	8.8%	0.52 [0.37, 0.72]
Chaiyakunapruk et al (2025) [29]	1291	34690	8468	130220	10.7%	0.57 [0.54, 0.61]
**Subtotal (95% CI)**	1443	**39692**	8821	**135275**	**50.2%**	**0.45 [0.35, 0.60]**
Heterogeneity: $Tau^2 = 0.08$; $Chi^2 = 17.70$, df = 5 (p< 0.01); $I^2 = 79\%$						
Test for overall effect: Z = -5.58 (p< 0.01)						
**No history of CVD, CKD, or heart failure**						
Simms-Williams et al (2024a) [43]	24	4875	50	4831	7.2%	0.48 [0.29, 0.77]
Simms-Williams et al (2024b) [42]	22	6526	66	6532	7.3%	0.33 [0.21, 0.54]
Lau et al (2022) [35]	38	1461	37	1427	7.6%	1.00 [0.64, 1.57]
Jensen et al (2020) [32]	23	1823	367	14841	7.9%	0.51 [0.34, 0.78]
Dave et al (2021) [30]	107	12584	129	12584	9.5%	0.83 [0.64, 1.07]
Jhu et al (2024) [33]	2635	61438	3181	60480	10.7%	0.82 [0.78, 0.86]
**Subtotal (95% CI)**	2849	**88707**	3830	**100695**	**49.8%**	**0.64 [0.46, 0.88]**
Heterogeneity: $Tau^2 = 0.12$; $Chi^2 = 23.15$, df = 5 (p< 0.01); $I^2 = 86\%$						
Test for overall effect: Z = -2.78 (p< 0.01)						
**Total (95% CI)**	4292	**128399**	12651	**235970**	**100%**	**0.53 [0.43, 0.67]**
Heterogeneity: $Tau^2 = 0.13$; $Chi^2 = 132.99$, df = 11 (p< 0.01); $I^2 = 95\%$						
Test for overall effect: Z = -5.37 (p< 0.01)						
Test for subgroup differences: $Chi^2 = 2.66$, df = 1 (p= 0.10)						

ESM Figure 6: Effect of combining a SGLT2 inhibitor and a GLP-1 RA compared to SGLT2 inhibitor or GLP-1 RA monotherapy on major adverse cardiovascular events stratified by risk of bias

Study	Combination therapy Events	Combination therapy Total	Monotherapy Events	Monotherapy Total	Weight	Risk ratio [95% CI]
**Low risk of bias**						
Lau et al (2022) [35]	38	1461	37	1427	8.7%	1.00 [0.64, 1.57]
**Subtotal (95% CI)**	38	**1461**	37	**1427**	**8.7%**	**1.00 [0.64, 1.57]**
Heterogeneity: $Tau^2 = 0.00$; $Chi^2 = 0.00$, df = 0 (p= 1.00); $I^2 = 0\%$						
Test for overall effect: Z = 0.01 (p= 0.99)						
**Moderate/serious/critical risk of bias**						
Marfella et al (2024) [39]	13	214	65	229	7.4%	0.21 [0.12, 0.38]
Jensen et al (2020) [32]	23	1823	367	14841	9.0%	0.51 [0.34, 0.78]
Liu et al (2025) [36]	40	208	63	208	9.8%	0.63 [0.45, 0.90]
Simms-Williams et al (2024a) [43]	45	6696	113	6696	9.8%	0.40 [0.28, 0.56]
Lopez et al (2022) [37]	45	343	87	343	10.0%	0.52 [0.37, 0.72]
Simms-Williams et al (2024b) [43]	55	8942	141	8942	10.2%	0.39 [0.29, 0.53]
Dave et al (2021) [30]	107	12584	129	12584	10.8%	0.83 [0.64, 1.07]
Jhu et al (2024) [33]	2635	61438	3181	60480	12.1%	0.82 [0.78, 0.86]
Chaiyakunapruk et al (2025) [29]	1291	34690	8468	130220	12.1%	0.57 [0.54, 0.61]
**Subtotal (95% CI)**	4254	**126938**	12614	**234543**	**91.2%**	**0.53 [0.41, 0.68]**
Heterogeneity: $Tau^2 = 0.12$; $Chi^2 = 129.23$, df = 8 (p< 0.01); $I^2 = 96\%$						
Test for overall effect: Z = -5.07 (p< 0.01)						
**Total (95% CI)**	4292	**128399**	12651	**235970**	**100%**	**0.56 [0.43, 0.71]**
Heterogeneity: $Tau^2 = 0.13$; $Chi^2 = 132.06$, df = 9 (p< 0.01); $I^2 = 96\%$						
Test for overall effect: Z = -4.63 (p< 0.01)						
Test for subgroup differences: $Chi^2 = 2.23$, df = 1 (p= 0.14)						

Risk ratio MH Random, 95% CI — axis: 0.1, 0.5, 1, 2, 10. Favours combination of SGLT-2 inhibitor and GLP-1 RA | Favours SGLT-2 inhibitor or GLP-1 RA monotherapy

ESM Figure 7: Effect of combining a SGLT2 inhibitor and a GLP-1 RA compared to SGLT2 inhibitor or GLP-1 RA monotherapy on major adverse cardiovascular events stratified by definition of MACE

Study	Combination therapy Events	Combination therapy Total	Monotherapy Events	Monotherapy Total	Weight	Risk ratio [95% CI]
**Three point MACE**						
Marfella et al (2024) [39]	13	214	65	229	7.4%	0.21 [0.12, 0.38]
Jensen et al (2020) [32]	23	1823	367	14841	9.0%	0.51 [0.34, 0.78]
Simms-Williams et al (2024a) [43]	45	6696	113	6696	9.8%	0.40 [0.28, 0.56]
Simms-Williams et al (2024b) [43]	55	8942	141	8942	10.2%	0.39 [0.29, 0.53]
Jhu et al (2024) [33]	2635	61438	3181	60480	12.1%	0.82 [0.78, 0.86]
Chaiyakunapruk et al (2025) [29]	1291	34690	8468	130220	12.1%	0.57 [0.54, 0.61]
**Subtotal (95% CI)**	4062	**113803**	12335	**221408**	**39.3%**	**0.47 [0.33, 0.66]**
Heterogeneity: $Tau^2 = 0.15$; $Chi^2 = 124.06$, df = 5 (p< 0.01); $I^2 = 98\%$						
Test for overall effect: Z = -4.36 (p< 0.01)						
**Other MACE**						
Lau et al (2022) [35]	38	1461	37	1427	8.7%	1.00 [0.64, 1.57]
Liu et al (2025) [36]	40	208	63	208	9.8%	0.63 [0.45, 0.90]
Lopez et al (2022) [37]	45	343	87	343	10.0%	0.52 [0.37, 0.72]
Dave et al (2021) [30]	107	12584	129	12584	10.8%	0.83 [0.64, 1.07]
**Subtotal (95% CI)**	230	**14596**	316	**14562**	**60.6%**	**0.71 [0.54, 0.93]**
Heterogeneity: $Tau^2 = 0.05$; $Chi^2 = 7.72$, df = 3 (p= 0.05); $I^2 = 61\%$						
Test for overall effect: Z = -2.45 (p= 0.01)						
**Total (95% CI)**	4292	**128399**	12651	**235970**	**100%**	**0.56 [0.43, 0.71]**
Heterogeneity: $Tau^2 = 0.13$; $Chi^2 = 132.06$, df = 9 (p< 0.01); $I^2 = 96\%$						
Test for overall effect: Z = -4.63 (p< 0.01)						
Test for subgroup differences: $Chi^2 = 2.81$, df = 1 (p= 0.09)						

Risk ratio MH Random, 95% CI — axis: 0.1, 0.5, 1, 2, 10. Favours combination of SGLT-2 inhibitor and GLP-1 RA | Favours SGLT-2 inhibitor or GLP-1 RA monotherapy

Three point MACE comprises cardiovascular death, non-fatal myocardial infarction, and non-fatal stroke

ESM Figure 8: Effect of combining a SGLT2 inhibitor and a GLP-1 RA compared to SGLT2 inhibitor or GLP-1 RA monotherapy on major adverse cardiovascular events stratified by method of adjusting for confounding

Study	Combination therapy Events	Combination therapy Total	Monotherapy Events	Monotherapy Total	Weight	Risk ratio [95% CI]
**Propensity scores/IPTW**						
Lau et al (2022) [35]	38	1461	37	1427	8.7%	1.00 [0.64, 1.57]
Liu et al (2025) [36]	40	208	63	208	9.8%	0.63 [0.45, 0.90]
Simms-Williams et al (2024a) [43]	45	6696	113	6696	9.8%	0.40 [0.28, 0.56]
Lopez et al (2022) [37]	45	343	87	343	10.0%	0.52 [0.37, 0.72]
Simms-Williams et al (2024b) [43]	55	8942	141	8942	10.2%	0.39 [0.29, 0.53]
Dave et al (2021) [30]	107	12584	129	12584	10.8%	0.83 [0.64, 1.07]
Jhu et al (2024) [33]	2635	61438	3181	60480	12.1%	0.82 [0.78, 0.86]
Chaiyakunapruk et al (2025) [29]	1291	34690	8468	130220	12.1%	0.57 [0.54, 0.61]
**Subtotal (95% CI)**	4256	**126362**	12219	**220900**	**83.5%**	**0.61 [0.49, 0.77]**
Heterogeneity: $Tau^2 = 0.09$; $Chi^2 = 113.77$, df = 7 ($p < 0.01$); $I^2 = 95\%$						
Test for overall effect: Z = -4.23 ($p < 0.01$)						
**Other method to adjust for confounding**						
Marfella et al (2024) [39]	13	214	65	229	7.4%	0.21 [0.12, 0.38]
Jensen et al (2020) [32]	23	1823	367	14841	9.0%	0.51 [0.34, 0.78]
**Subtotal (95% CI)**	36	**2037**	432	**15070**	**16.4%**	**0.34 [0.14, 0.79]**
Heterogeneity: $Tau^2 = 0.31$; $Chi^2 = 5.86$, df = 1 ($p = 0.02$); $I^2 = 83\%$						
Test for overall effect: Z = -2.50 ($p = 0.01$)						
**Total (95% CI)**	4292	**128399**	12651	**235970**	**100%**	**0.56 [0.43, 0.71]**
Heterogeneity: $Tau^2 = 0.13$; $Chi^2 = 132.06$, df = 9 ($p < 0.01$); $I^2 = 96\%$						
Test for overall effect: Z = -4.63 ($p < 0.01$)						
Test for subgroup differences: $Chi^2 = 3.28$, df = 1 ($p = 0.07$)						

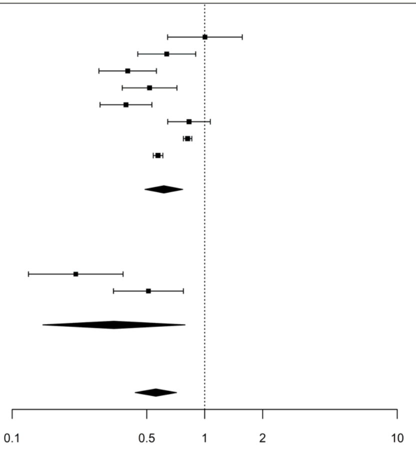

ESM Figure 9: Effect of combining a SGLT2 inhibitor and a GLP-1 RA compared to SGLT2 inhibitor or GLP-1 RA monotherapy on major adverse cardiovascular events stratified by data source

Study	Combination therapy Events	Combination therapy Total	Monotherapy Events	Monotherapy Total	Weight	Risk ratio [95% CI]
**Electronic healthcare records**						
Lau et al (2022) [35]	38	1461	37	1427	8.7%	1.00 [0.64, 1.57]
Jensen et al (2020) [32]	23	1823	367	14841	9.0%	0.51 [0.34, 0.78]
Liu et al (2025) [36]	40	208	63	208	9.8%	0.63 [0.45, 0.90]
Simms-Williams et al (2024a) [43]	45	6696	113	6696	9.8%	0.40 [0.28, 0.56]
Simms-Williams et al (2024b) [43]	55	8942	141	8942	10.2%	0.39 [0.29, 0.53]
Jhu et al (2024) [33]	2635	61438	3181	60480	12.1%	0.82 [0.78, 0.86]
**Subtotal (95% CI)**	2836	**80568**	3902	**92594**	**59.6%**	**0.59 [0.43, 0.80]**
Heterogeneity: $Tau^2 = 0.12$; $Chi^2 = 43.34$, df = 5 ($p < 0.01$); $I^2 = 87\%$						
Test for overall effect: Z = -3.40 ($p < 0.01$)						
**Other data sources**						
Marfella et al (2024) [39]	13	214	65	229	7.4%	0.21 [0.12, 0.38]
Lopez et al (2022) [37]	45	343	87	343	10.0%	0.52 [0.37, 0.72]
Dave et al (2021) [30]	107	12584	129	12584	10.8%	0.83 [0.64, 1.07]
Chaiyakunapruk et al (2025) [29]	1291	34690	8468	130220	12.1%	0.57 [0.54, 0.61]
**Subtotal (95% CI)**	1456	**47831**	8749	**143376**	**40.3%**	**0.50 [0.30, 0.83]**
Heterogeneity: $Tau^2 = 0.24$; $Chi^2 = 20.08$, df = 3 ($p < 0.01$); $I^2 = 94\%$						
Test for overall effect: Z = -2.67 ($p < 0.01$)						
**Total (95% CI)**	4292	**128399**	12651	**235970**	**100%**	**0.56 [0.43, 0.71]**
Heterogeneity: $Tau^2 = 0.13$; $Chi^2 = 132.06$, df = 9 ($p < 0.01$); $I^2 = 96\%$						
Test for overall effect: Z = -4.63 ($p < 0.01$)						
Test for subgroup differences: $Chi^2 = 0.27$, df = 1 ($p = 0.61$)						

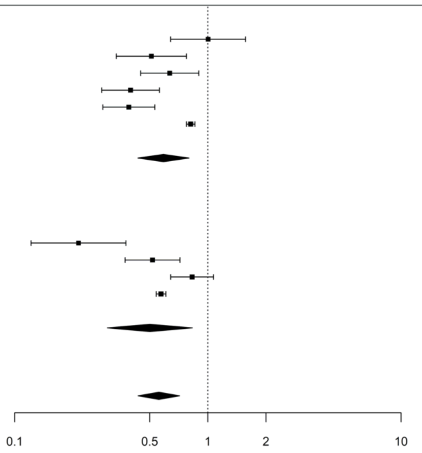

Supplement: Supplementary file 1 — ESM (PDF 3212 KB) [file 125_2025_6565_MOESM1_ESM.pdf]
